# Hierarchical Strain‐Modified Medium‐Entropy Carbide Ceramics Exhibit Exceptional Ablation Resistance up to 2400°C

**DOI:** 10.1002/advs.202518785

**Published:** 2026-01-28

**Authors:** Junyi Xiao, Pengfei He, Lin Xue, Shujun Hu, Liliang Shao, Chuan Sun, Yunyun Ge, Jiangbo Cheng, Xiubing Liang

**Affiliations:** ^1^ College of Materials Science and Engineering Hohai University Changzhou P. R. China; ^2^ Defense Innovation Institute Academy of Military Sciences Beijing P. R. China

**Keywords:** ablation resistance, first‐principles calculations, hierarchical strain modulation, medium entropy carbides, ultrahigh‐temperature ceramics

## Abstract

Carbide ultrahigh‐temperature ceramics (UHTCs) exhibit high melting points and are regarded as promising candidate materials for applications in ultrahigh‐temperature conditions, such as high‐speed flight vehicles. However, a high melting point alone is inadequate because thermal shock is typically associated with intense shear and oxidation, which require carbide UHTCs to not only have elevated temperature resistance but also robust structural stability. Herein, we present a reduced graphene oxide (rGO)‐reinforced (HfZrTi)C medium‐entropy ceramic (HZTMEC) that maintains structural integrity under thermal shock up to 2400°C and forms a dense and flat oxide layer. Notably, the addition of rGO induced a hierarchical strain modification spanning multiple length scales. At the microscale, rGO doping enhances atomic strain, refines grain size, and expands the distribution of high‐strain regions, increasing dislocation density and hindering dislocation movement. At the mesoscale, the oxidation and volatilization of rGO during ablation create strip‐shaped micropores in the oxide layer, dispersing the transformation stress and thermal stress generated by thermal shock. Therefore, long‐term thermal stability without fracture over 2400°C was achieved in UHTCs. This study provides a valuable strategy for balancing ultrahigh‐temperature ablation resistance and structural stability.

## Graphical abstract

A novel strategy is developed by doping reduced graphene oxide (rGO) into a (HfZrTi)C medium‐entropy ceramic. This induces a hierarchical strain modification mechanism, synergistically inhibiting crack propagation at the microscale and dissipating stress at the mesoscale. The resulting composite maintains structural integrity under thermal‐mechanical‐oxygen coupling ablation, showing great potential for long‐term service up to 2400°C.

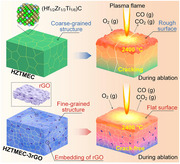

## 1 Introduction

The increase in Mach numbers for high‐speed flight vehicles imposes increasingly stringent requirements on thermal protection systems. Currently, thermal protection requirements are geared toward ultrahigh‐temperature environments over 2000°C [1, 2]. Owing to their exceptional melting points and thermal stability, carbide ultrahigh‐temperature ceramics (UHTCs) are uniquely positioned to meet these extreme demands [3, 4, 5]. However, thermal shock in ultrahigh‐temperature environments is often coupled with strong shear and oxidation effects, which requires carbide UHTCs and their oxidation products to not only have a high‐temperature limit but also maintain structural stability during service. Unfortunately, traditional carbide UHTCs often undergo brittle fracture under thermal‐mechanical‐oxidative coupled thermal shocks, limiting their practical applications.

This bottleneck stems from the failure behavior of traditional carbide UHTCs at multiple scales. On the mesoscopic scale, the ablation‐formed surface oxide layer is the first defense against thermal shock, but transformation and thermal stresses accumulate there without effective dissipation, producing cracks [6]. On the microscopic scale, the underlying carbide ceramic is intrinsically brittle and lacks mechanisms to suppress crack initiation and propagation [7]. Upon initiation, cracks rapidly propagate inward, leading to catastrophic failure. This cross‐scale failure mechanism constitutes a key limitation to the broader application of carbide UHTCs.

Recent studies have improved the mechanical properties of carbide UHTCs by introducing toughening phases or high‐entropy strategies [8, 9, 10, 11]. Carbon fibers and SiC are effective toughening phases [12, 13, 14]. The former inhibits crack propagation through pull‐out behavior, whereas the latter improves fracture toughness (*K_IC_*) by inducing crack deflection and bridging. Unfortunately, although carbon fibers and SiC inhibited crack propagation at the microscopic scale, they negatively affected the structural integrity of the oxide layer at the mesoscopic scale. Because of the morphology and defects in the oxidation resistance of the carbon fibers, the oxide layer was covered with groove‐like morphologies and tubular pores after ablation [15, 16]. SiC oxidation forms SiO_2_ phases with a low melting point (approximately 1723°C), which causes severe loss in ablation above 2000°C, resulting in a structurally loose oxide layer that cannot withstand thermal shock [16, 17]. Therefore, these strategies primarily focus on the microscale, neglecting the structural integrity of the oxide layer at the mesoscopic scale. Similarly, high‐entropy strategies improve material properties at both atomic and nanoscale levels by introducing multiple elements [18, 19, 20, 21]. However, the oxidation products of some elements have low melting points (∼1520°C for Nb_2_O_5_ and ∼1473°C for WO_3_) and are also prone to excessive volatilization, leading to the fracture of the oxide layer [22, 23].

Distinct from high‐entropy strategies, medium‐entropy ceramics retaining only a portion of effective elements have garnered significant attention, with the (HfZrTi)C medium‐entropy ceramic (HZTMEC) being a prime composition [24, 25, 26]. The metal elements in HZTMEC all belong to Group IVB, which not only facilitates single‐phase synthesis but also lays the foundation for forming dense oxides and enhancing ablation resistance. Related research further enhanced performance by combining (HfZrTi)C with various constituents, including SiC, carbon fibers, and C/C composites, thereby achieving ultrahigh‐temperature ablation resistance around 2000–2200°C [17, 24, 25, 26]. However, these strategies still struggle to prevent cracking of the oxide layer [15, 16, 25, 26]. Thus, simultaneously maintaining the structural integrity of the oxide layer while achieving ultrahigh‐temperature ablation resistance remains a significant challenge.

To overcome these bottlenecks, we propose a hierarchical strain modulation strategy via the incorporation of 3 vol% reduced graphene oxide (rGO) into HZTMEC, forming the HZTMEC‐3rGO. Departing from existing strategies, the innovation here lies in utilizing rGO as a multifunctional phase that serves as both a toughening and a sacrificial phase capable of dissipating thermal shock stress. Therefore, the hierarchical strain modulation proposed here involves not only an inhibition mechanism for crack propagation at the microscale but also a stress dissipation mechanism at the mesoscale. Specifically, HZTMEC‐3rGO passes the ablation testing at temperatures up to 2400°C for 240 s. The mass and linear ablation rates for HZTMEC‐3rGO were significantly reduced by 22.4% and 43.9%, respectively, compared with those of HZTMEC. HZTMEC‐3rGO possesses a finer grain size and forms a dense and flat oxide layer. Meanwhile, its *K_IC_* reaches 7.514 MPa·m^1/2^. More importantly, HZTMEC‐3rGO simultaneously maintains the structural integrity of the oxide layer and exhibits ultrahigh‐temperature ablation resistance in thermal‐mechanical‐oxidative coupled ablation environments. This hierarchical strain‐modulation paradigm offers new insights for developing advanced thermal‐protection ceramics.

## 2 Results and Discussion

### 2.1 Design Strategy and Microstructure Analysis

**FIGURE 1:**
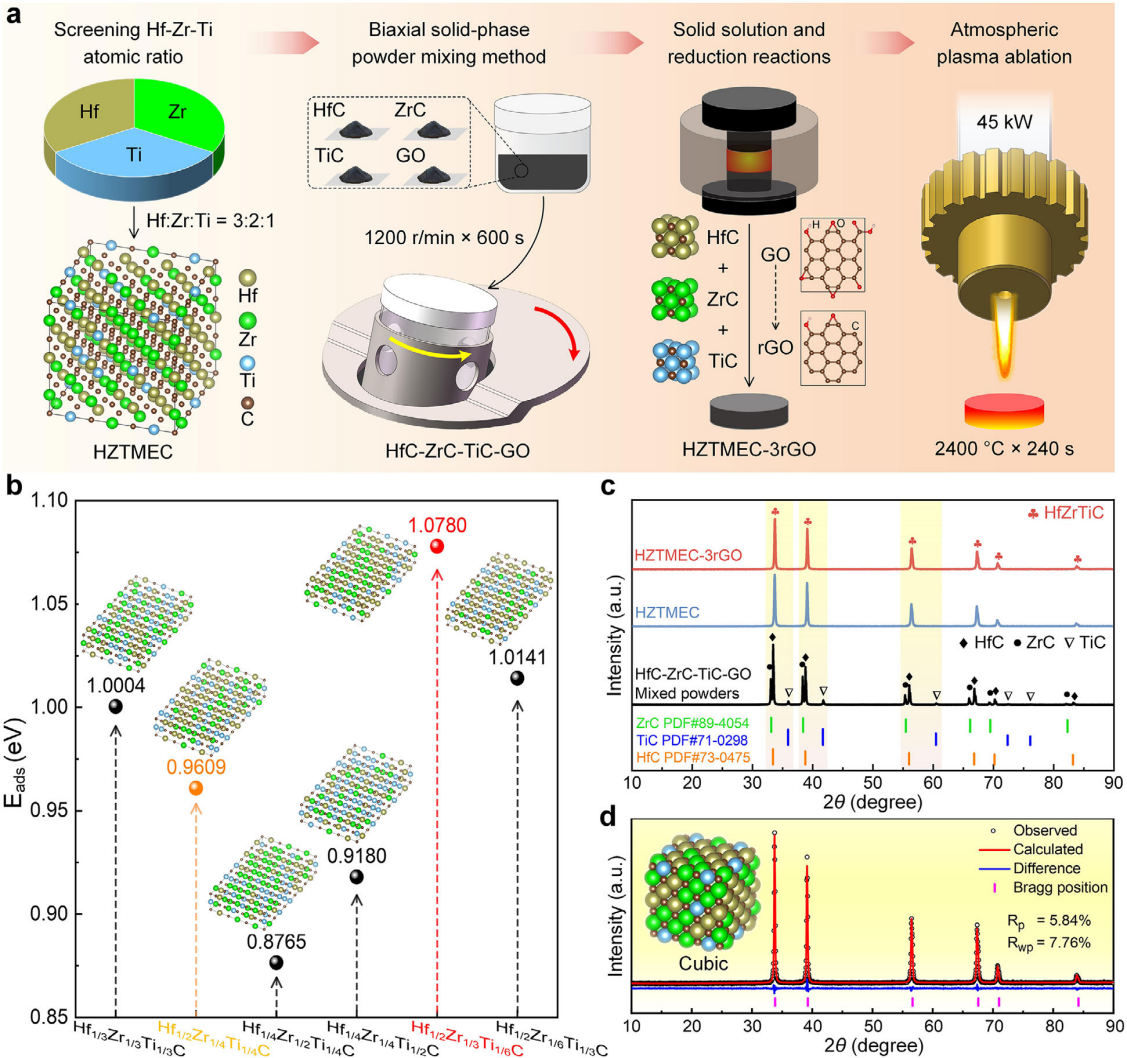
Design strategy, fabrication, and ablation of HZTMEC‐3rGO. (a) Design strategy: First‐principles screening of Hf‐Zr‐Ti atomic ratios, biaxial solid‐phase mixing for powder preparation, solid solution synthesis of HZTMEC with simultaneous in situ reduction of rGO, and plasma ablation testing. (b) Oxygen adsorption energy barriers for HZTMEC at different atomic ratios, highlighting the optimal Hf:Zr: Ti = 3:2:1 configuration, and the insets showing the corresponding atomic model. (c) XRD patterns of the original mixed powder, HZTMEC, and HZTMEC‐3rGO, confirming single‐phase solid solution formation. (d) Rietveld refinement of the HZTMEC XRD pattern, with inset showing the unit cell structure schematic.

Figure 1 includes the design strategy, fabrication, and ablation of HZTMEC‐3rGO. Figure 1a illustrates the design strategy for hierarchical strain construction by doping HZTMEC with rGO. This strategy comprises four key stages: (i) screening the optimal Hf‐Zr‐Ti atomic ratio via first‐principles calculations, (ii) preparing original powders using the biaxial solid‐phase mixing method, (iii) synthesizing HZTMEC via a solid solution reaction while achieving the in situ reduction of rGO, and (iv) evaluating the performance via plasma ablation testing.

First‐principles calculations (Figure 1b) revealed the oxygen atom adsorption energy barriers on the (100) crystal plane for various Hf‐Zr‐Ti atomic ratios. In the models with Hf‐Zr‐Ti atomic ratios of 1:1:1 and 2:1:1, the oxygen adsorption energy of (Hf_1/3_Zr_1/3_Ti_1/3_)C was the highest (1.0004 eV), followed by that of (Hf_1/2_Zr_1/4_Ti_1/4_)C (approximately 0.9609 eV). The values of (Hf_1/4_Zr_1/2_Ti_1/4_)C and (Hf_1/4_Zr_1/4_Ti_1/2_)C were relatively low. Because of the high oxygen adsorption energy barrier of (Hf_1/2_Zr_1/4_Ti_1/4_)C, two additional models were calculated, i.e., (Hf_1/2_Zr_1/3_Ti_1/6_)C and (Hf_1/2_Zr_1/6_Ti_1/3_)C. The (Hf_1/2_Zr_1/3_Ti_1/6_)C model (Hf:Zr: Ti = 3:2:1) exhibited a favorable O‐adsorption barrier of 1.078 eV and was therefore selected as the synthesis route for HZTMEC in this study. Herein, during synthesis, the reduction of GO was integrated with the sintering of HZTMEC to enable the in situ generation of rGO within the ceramic matrix. The amounts of rGO are 1, 3, and 5 vol% based on the existing relevant literature [27, 28], and the data for HZTMEC‐1rGO and HZTMEC‐5rGO are provided in the supplementary materials.

Figure 1c shows the X‐ray diffraction (XRD) patterns before and after sintering, illustrating the formation of a single‐phase solid solution. The characteristic peaks of GO and rGO were not observed in the XRD patterns, consistent with their low mass fractions. The value of 3 vol% corresponds to a mass fraction of 0.73 wt% in HZTMEC‐3rGO, which is difficult to detect by conventional XRD. Therefore, subsequent characterization provided further evidence for rGO addition. Furthermore, Figure 1d shows the Rietveld refinement results for the HZTMEC, in which the R‐pattern (R_p_) and R‐weighted pattern (R_wp_) were 5.84% and 7.76%, respectively. Both values are within the acceptable range, confirming the reliability of the refinement results. The crystal structure parameters of HZTMEC are listed in Table S1. From Figure 1c–d and Table S1, HZTMEC is confirmed to be FCC with the space group $\mathrm{Fm}\bar{3}m$ and similar to HfC (PDF#73‐0475). This implies that HZTMEC can be regarded as a substitution solid solution, in which the cation sites occupied by Hf atoms in the crystal lattice are partially replaced by Zr and Ti atoms. The ideal unit cell structure is shown in the inset of Figure 1d.

**FIGURE 2:**
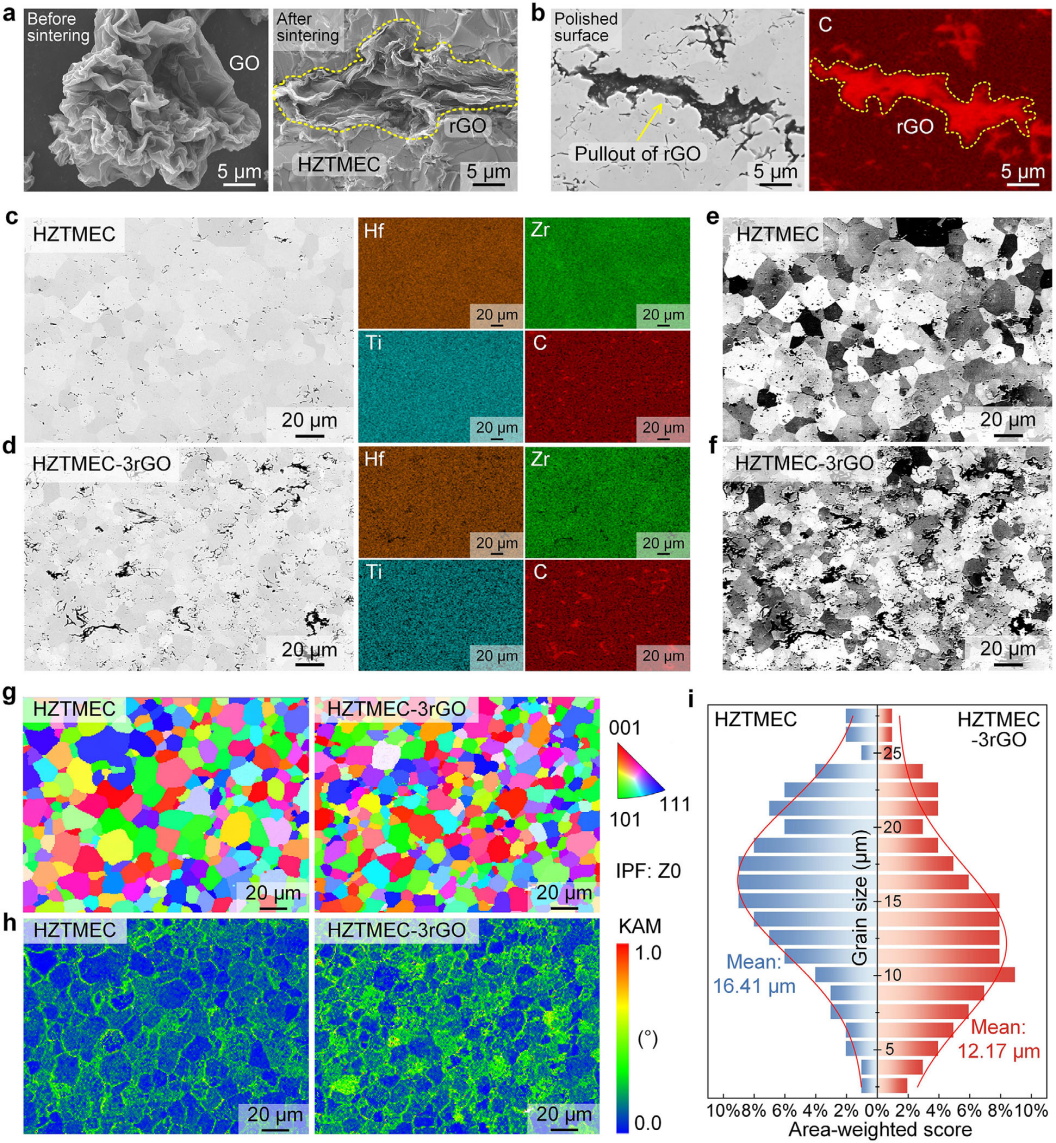
Morphology and strain analysis at the micrometer scale. (a) Original morphology of GO before sintering and the morphology of rGO doped in HZTMEC after sintering. (b) rGO in the polished surface and its C element distribution, showing strip‐shaped micropores left after rGO pullout. (c, d) SEM images and EDS maps of HZTMEC and HZTMEC‐3rGO. (e, f) BSE images of HZTMEC and HZTMEC‐3rGO, preliminarily showing grain refinement of HZTMEC after rGO doping. (g–i) IPF, KAM, and grain size statistics of HZTMEC and HZTMEC‐3rGO.

To demonstrate the addition of rGO to HZTMEC and its effects, morphological and strain analyses at the micrometre scale were conducted by scanning electron microscopy (SEM), and the results are shown in Figure 2. During the sintering of the HZTMEC, GO can undergo a simultaneous reduction reaction, achieving the in situ generation of rGO, which is manifested as the desorption of the O atom in GO. The energy‐dispersive spectroscopy (EDS) results in Figure S1 show that the percentage of O atoms decreased from approximately 35–36 at.% before sintering to approximately 1–2 at.% after sintering. Regarding the morphology, the microstructure of GO in Figure 2a resembles that of a paper ball, whereas rGO exhibits a layered, sheet‐like morphology. This was attributed to the combined effects of GO agglomeration, hot‐pressing, and grain growth of HZTMEC, indicating the successful addition of rGO to HZTMEC. In addition, strip‐shaped micropores enriched with C were observed on the polished surface of the sample (Figure 2b), with lengths similar to those of the sheet‐like rGO in Figure 1a. Therefore, it is reasonable to speculate that these pores were formed owing to the pulling‐out behavior of rGO during polishing, with residual rGO still present.

Figure 2c, d shows the SEM images and EDS maps of HZTMEC and HZTMEC‐3rGO. The distribution of all the elements is uniform, with no obvious segregation. The EDS results (Figure S2) confirm that the atomic ratio of Hf‐Zr‐Ti was the target ratio of 1/2:1/3:1/6. Among them, Hf and Zr exhibited compositional fluctuations, whereas Ti remained relatively stable. This is due to the differences in the atomic radius and high‐temperature diffusion. Although Ti, Zr, and Hf belong to the same Group IVB, the atomic radius of Ti is significantly smaller than that of Zr and Hf. This gives Ti a stronger high‐temperature diffusion capability, which facilitates the homogenization of the composition distribution. Compared to Ti, Zr, and Hf have larger atomic radii and lower atomic diffusion coefficients. This indicates that during sintering, Zr and Hf are more prone to compositional segregation, leading to compositional fluctuations. As shown in Figure 2d, strip‐shaped micropores, analogous to those observed in Figure 2b, are distributed throughout the sample. The concurrent enrichment of carbon within these micropores confirms the dispersion of rGO in the HZTMEC matrix. Figure 2e,f present the backscattered electron (BSE) images corresponding to Figure 2c,d, revealing grain refinement in HZTMEC‐3rGO.

To further quantify the grain refinement phenomenon, Figure 2g–i shows the electron backscatter diffraction (EBSD) results for HZTMEC and HZTMEC‐3rGO, including the inverse pole figure (IPF), kernel average misorientation (KAM), and grain size statistics. The IPF in Figure 2g reveals a refined grain structure in HZTMEC‐3rGO compared to that in HZTMEC. The statistics in Figure 2i show that the average grain size of HZTMEC is 16.41 µm, whereas HZTMEC‐3rGO is 12.17 µm (reduced by about 4 µm), which is refined by 25.84%. Figure 2h shows the KAM values of HZTMEC and HZTMEC‐3rGO. The main difference between the two lies in the distribution region of high KAM values. In HZTMEC, it is primarily concentrated at the grain boundaries. However, in HZTMEC‐3rGO, the distribution extends beyond the grain boundaries to a pervasive presence within the grain interiors, covering a significantly broader area. This indicates that the grain refinement caused by rGO doping results in more grain boundary strain and causes obvious strain within the grains. Ultimately, it expands the distribution of the high‐strain regions in HZTMEC‐3rGO. Furthermore, this implies a high‐density dislocation distribution, making it easier for dislocations to pin and entangle, hindering dislocation movement. Therefore, the improved mechanical properties contributed by rGO addition fundamentally originate from its effective modulation of strain distribution at the micrometer scale, encompassing both grain boundaries and interiors.

To illustrate the differences in rGO content, Figure S3 shows SEM and BSE images of HZTMEC‐5rGO. The grain refinement observed for HZTMEC‐5rGO was similar to that observed for HZTMEC‐3rGO without any further refinement. Furthermore, Figure S4 indicates that obvious agglomeration occurred in HZTMEC‐5rGO; the agglomeration area significantly increased from 1.268% in HZTMEC‐3rGO to 3.763% in HZTMEC‐5rGO. Therefore, a higher rGO content is not necessarily better. Considering that the addition of rGO inevitably introduces micropores, we tested the densities and thermal conductivities of HZTMEC, HZTMEC‐3rGO, and HZTMEC‐5rGO; the results are shown in Figure S5. As rGO content increased, both density and thermal conductivity decreased. The density drop aligns with our earlier inference, while the thermal‐conductivity drop reflects competing effects. Despite the high intrinsic conductivity of rGO, the increased porosity, grain refinement, and lattice distortion collectively shorten the phonon mean free path, resulting in an overriding reduction in thermal conductivity.

**FIGURE 3:**
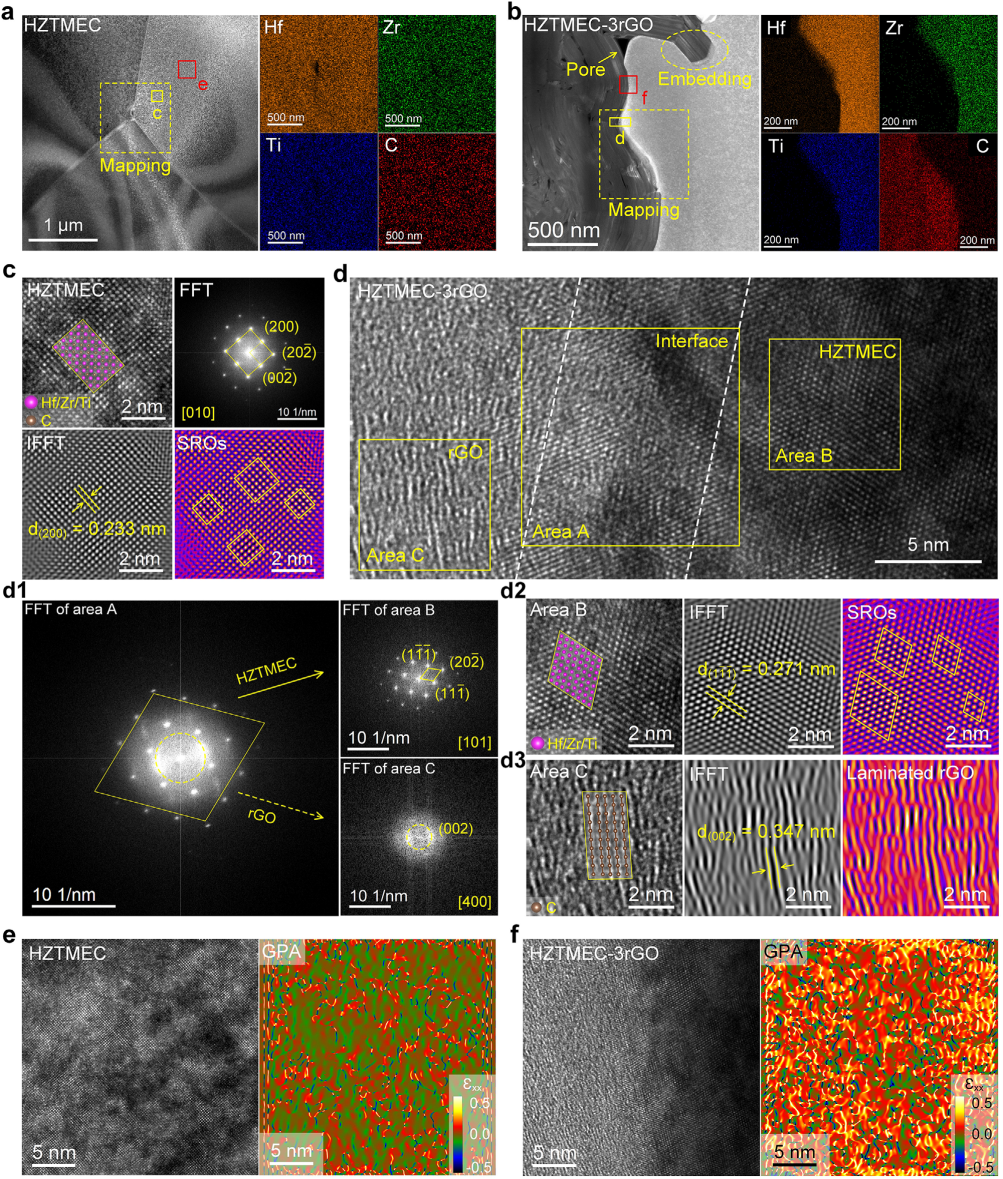
Phase and strain analysis at the nanoscale. (a,b) DF images and local EDS maps of HZTMEC and HZTMEC‐3rGO. (c) HRTEM image of area c in Figure a (inset shows the corresponding atomic arrangement of HZTMEC), FFT pattern, IFFT pattern, and a highlighted view of the IFFT pattern (highlighting the SROs region). (d) HRTEM image of area d in Figure b, where area A is the interface between HZTMEC and rGO, and areas B and C are HZTMEC and rGO, respectively. (d1) FFT patterns of areas A, B, and C. (d2) HRTEM image, IFFT pattern, and corresponding highlighted image of area B. (d3) HRTEM image of area C (inset showing the corresponding atomic arrangement of rGO), IFFT pattern, and corresponding highlighted image (highlighting the layered morphology of rGO). (e) HRTEM image and GPA of area e (in Figure 3a), showing lower atomic strain in HZTMEC. (f) HRTEM image and GPA of area f (in Figure 3b), showing higher atomic strain in HZTMEC‐3rGO.

To further analyze the influence of rGO, Figure 3 presents the characterized results of phase and strain at the nanoscale level. Figure 3a shows a dark‐field (DF) image of HZTMEC, revealing the intersection region of the three equiaxed crystal boundaries. EDS mapping revealed that Hf, Zr, Ti, and C were uniformly distributed, confirming the completion of the solid–solution reaction at the nanoscale level. Figure 3b shows a DF image of HZTMEC‐3rGO, which exhibits a morphology with light and dark phases. Combining the EDS mapping, the dark phase was identified as rGO, and the light phase was HZTMEC. The doped rGO phase shows a multilayered stacking morphology and introduces pores into the HZTMEC matrix, validating the previous assertion that rGO addition inevitably creates porosity. Figure S6 shows the distribution of rGO, which was primarily at the grain boundaries of the HZTMEC and exhibited an embedded morphology (Figure S6a). This also suggests that rGO is compressed by the growing HZTMEC grains (Figure S6b), with clear interfaces (Figure S6c). Figure 3c demonstrates the further characterization of area c in Figure 3a. By combining the fast Fourier transform (FFT) pattern and Table S1, the calibration of the HZMMEC was completed. The crystal plane indices corresponding to the low‐index diffraction spots were (200), $\left(20\bar{2}\right)$, and $\left(00\bar{2}\right)$, with the zone axis being [010] and the lattice fringe spacing of (200) being 0.233 nm. The corresponding atomic arrangement is presented in the inset of the high‐resolution transmission electron microscopy (HRTEM) image in Figure 3a. In addition, bright regions (within the rectangular boxes) were identified in the highlighted image modified by the inverse fast Fourier transform (IFFT). Based on the principles of transmission electron microscopy (TEM), these regions can be regarded as aggregates of Hf atoms with high atomic numbers, which are regarded as short‐range ordering (SROs) within the crystal lattice [29, 30].

An enlarged HRTEM image of area d in Figure 3b is shown in Figure 3d. The interface between the HZTMEC and rGO was distinguished by the light and dark regions (area A). In Figure 3b, the dark phase (B) corresponds to the HZTMEC matrix, while the light phase (C) is identified as the rGO. Figure 3d, 1 shows the FFT patterns for areas A, B, and C. The FFT pattern of area A exhibited two sets of diffraction spots, corresponding to the FFT patterns of areas B and C, confirming the addition of rGO at the atomic scale. In the FFT of area B, the low‐index diffraction spots had crystal plane indices of $\left(1\bar{1}\bar{1}\right)$, $\left(20\bar{2}\right)$, and $\left(11\bar{1}\right)$, and the zone axis was [101]. In the FFT of area C, a weakly amorphous phenomenon was observed, with diffraction spots corresponding to the (002) crystal plane and a zone axis of [400]. This indicates that rGO is perpendicular to the observation plane, and multilayer stacking and compression distortion lead to a weak amorphous structure. Figure 3d, 2,d3 shows the HRTEM, IFFT, and highlighted images of areas B and C in Figure 3d, respectively. The IFFT spectrum in Figure 3d, 2 shows a lattice fringe spacing of 0.271 nm corresponding to the $\left(1\bar{1}\bar{1}\right)$ crystal plane and the presence of SROs (within the diamond‐shaped boxes). This confirms that this region is HZTMEC, with the corresponding atomic arrangement shown in the inset of the HRTEM image. In Figure 3d3, the measured interplanar spacing of 0.347 nm corresponds to the (002) plane. This attribution is further confirmed by the IFFT image, which reveals the multilayer stacking of rGO with compressive distortion.

Regarding atomic strain, Figure 3e,f show the geometrical phase analysis (GPA) results for HZTMEC and HZTMEC‐3rGO, respectively, with the selected areas being area e (in Figure 3a) and area f (in Figure 3b). Although strain distributions in both materials were relatively uniform, the atomic strain in HZTMEC‐3rGO was significantly higher than that in HZTMEC. A higher atomic strain implies a higher dislocation density, which is confirmed by the IFFT patterns in Figure S7, and is consistent with the KAM results in Figure 2h. Overall, rGO addition induces nanoscale atomic strain and increases dislocation density, promoting entanglement that hinders dislocation motion and improves mechanical properties. Figure 3f and Figure S7 clarify how rGO increases atomic strain in HZTMEC‐3rGO, i.e., rGO introduces heterointerfaces (Figure 3f) and additional microdefects, such as dislocations (Figure S7), which displace atoms from ideal lattice sites and raise the strain.

### 2.2 Ablation Behavior

**FIGURE 4:**
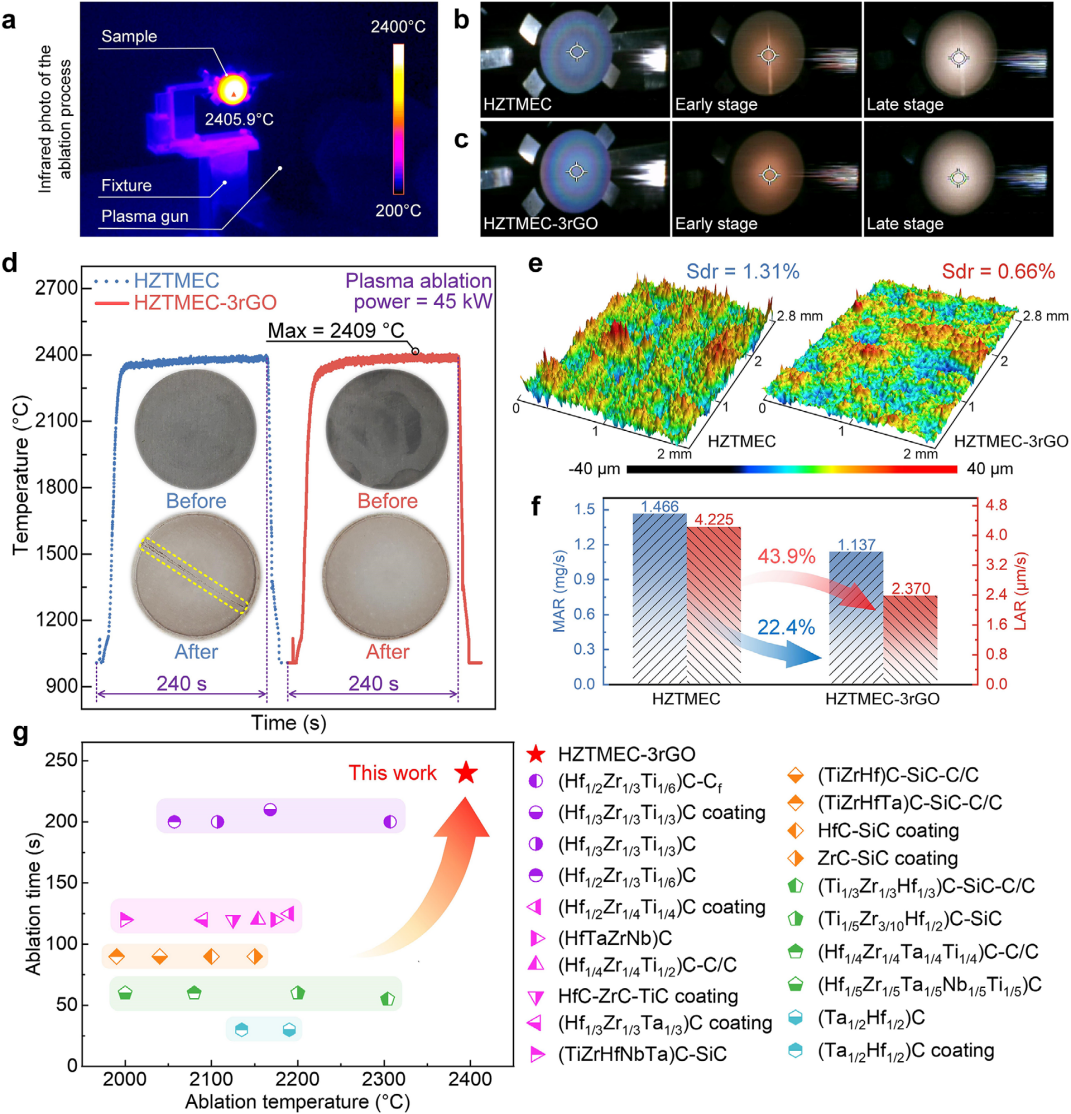
Ablation performance of HZTMEC and HZTMEC‐3rGO. (a) Infrared photo of HZTMEC‐3rGO at the high‐temperature stage. (b, c) Photographs of HZTMEC and HZTMEC‐3rGO at the stages of plasma flame ignition and the early and late ablation, showing obvious cracking of HZTMEC and intact HZTMEC‐3rGO. (d) Ablation curves of HZTMEC and HZTMEC‐3rGO, with photographs of the two before and after ablation shown in the inset. (e) 3D surface morphology of HZTMEC and HZTMEC‐3rGO after ablation. (f) MAR and LAR of HZTMEC and HZTMEC‐3rGO, demonstrating the improvement in ablation rate due to rGO doping. (g) Comparison between this study and a series of existing similar studies, showing the advantages of HZTMEC‐3rGO in terms of ablation temperature and time [15, 16, 25, 26, 36–48].

Ablation testing is a key method for evaluating the quality of UHTCs and involves indexes such as the ablation temperature, ablation time, mass ablation rate (MAR), and linear ablation rate (LAR). The ablation performances of HZTMEC and HZTMEC‐3rGO are shown in Figure 4. Figure 4a presents an infrared photograph of HZTMEC‐3rGO during high‐temperature testing. The sample appears in a high‐brightness state due to thermal shock from the plasma flame, with a recorded central temperature reaching 2405.9°C. Figure 4b,c show photographs of the HZTMEC and HZTMEC‐3rGO at plasma flame ignition and the stages of early and late ablation, respectively. At the plasma ignition stage, both the HZTMEC and HZTMEC‐3rGO remained intact. However, in the early and late ablation stages, the HZTMEC exhibited brittle cracking. This phenomenon is commonly observed in bulk ceramics during ablation tests. Notably, unlike HZTMEC, HZTMEC‐3rGO exhibited no cracking during ablation. These results indicate that HZTMEC‐3rGO has superior mechanical properties, supporting the earlier conclusion that rGO‐induced strain modulation indirectly enhances mechanical performance.

Figure 4d presents the ablation curves of HZTMEC and HZTMEC‐3rGO, exhibiting an ablation process sustained for 240 s with temperatures reaching up to 2400°C (with a peak temperature of 2409°C). The insets show the corresponding samples before and after ablation, revealing distinct cracking in HZTMEC, whereas HZTMEC‐3rGO maintains an intact surface. These results demonstrate that HZTMEC‐3rGO passed the ablation testing at temperatures up to 2400°C and 240 s. Additionally, HZTMEC‐1rGO and HZTMEC‐5rGO were subjected to the same ablation testing (Figure S8). Similar to HZTMEC‐3rGO, HZTMEC‐1rGO maintained the integrity of its structure and oxide layer, while HZTMEC‐5rGO exhibited brittle fracture similar to that of HZTMEC. Single‐edge notched beam (SENB) tests were conducted on HZTMEC, HZTMEC‐1rGO, HZTMEC‐3rGO, and HZTMEC‐5rGO. The results are shown in Figure S9. The *K_IC_* values for HZTMEC, HZTMEC‐1rGO, HZTMEC‐3rGO, and HZTMEC‐5rGO were 6.956, 7.017, 7.514, and 6.857 MPa·m^1/2^, respectively, which are consistent with the ablation results. This indicates that 3 vol% was the optimal amount of rGO added to this experimental system. Excessive rGO led to more severe agglomeration and excessive pores (Figure S3–S5), which eventually deteriorated toughening.

To explain the effect of rGO addition on *K_IC_*, Equation (4) was obtained by combining Equations (1), (2), and (3): Equation (1) was applied to calculate the *K_IC_* of solid ceramics, whereas Equations (2) and (3) present the relationships between the bulk modulus (*B*), shear modulus (*G*), elastic modulus (*E*), and Poisson's ratio (*v*): [31]

(1) $$K_{IC}={V_{0}}^{1/6}G{\left(\frac{B}{G}\right)}^{1/2}$$

(2) $$G=\frac{E}{2\left(1+\nu \right)}$$

(3) $$B=\frac{E}{3\left(1-2\nu \right)}$$

(4) $$K_{IC}={V_{0}}^{1/6}G{\left(\frac{2\left(1+\nu \right)}{3\left(1-2\nu \right)}\right)}^{1/2}$$

where *V_0_* denotes the volume per atom. In Equation (4), *K_IC_* is positively correlated with *V_0_*, *G*, and *v*. The effects of graphene addition on *G* and *v* were confirmed; the former increased with the addition of graphene, whereas the latter decreased with the addition of graphene [32, 33]. To investigate the effect of the rGO addition on *V_0_*, a relevant atomic model was established (Figure S10). In the modeling, rGO was idealized as graphene with a layer spacing of approximately 0.34 nm, and the primary exposed crystal plane of HZTMEC was set as (100) [34, 35]. By first‐principles calculations, the *V_0_* of HZTMEC is 12.35 Å^3^, while that of HZTMEC‐3rGO is 13.17 Å^3^. This indicates that doping rGO can enhance *K_IC_* by increasing *V_0_*. However, excessive rGO may reduce *K_IC_* by decreasing *v*. And if excess rGO introduces pores due to agglomeration, it decreases *G*, resulting in reduced *K_IC_*. Concurrently, an optimal rGO content of ∼3 vol% was identified, as derived from the relationship between *K_IC_* and rGO content plotted in Figure S11.

Furthermore, this study analyzed the 3D morphology of the ablated surfaces of HZTMEC and HZTMEC‐3rGO (Figure 4e) and calculated the MAR and LAR (Figure 4f). As shown in Figure 4e, the Sdr of HZTMEC and HZTMEC‐3rGO after ablation were 1.31% and 0.66%, respectively. This indicates that the ablation surface of HZTMEC‐3rGO was flatter and better resisted the thermal shock of the plasma flame, whereas HZTMEC exhibited a rougher surface, corresponding to deeper ablation by the plasma flame. As shown in Figure 4f, compared to HZTMEC, the MAR and LAR of HZTMEC‐3rGO were significantly reduced by 22.4% and 43.9%, respectively, consistent with the results in Figure 4e. Finally, to fully demonstrate the excellent ablation resistance of HZTMEC‐3rGO, this study compared the results of HZTMEC‐3rGO with a series of similar studies (Figure 4g), highlighting the significant advantages of HZTMEC‐3rGO in terms of ablation temperature and time.

### 2.3 Ablation Mechanisms

**FIGURE 5:**
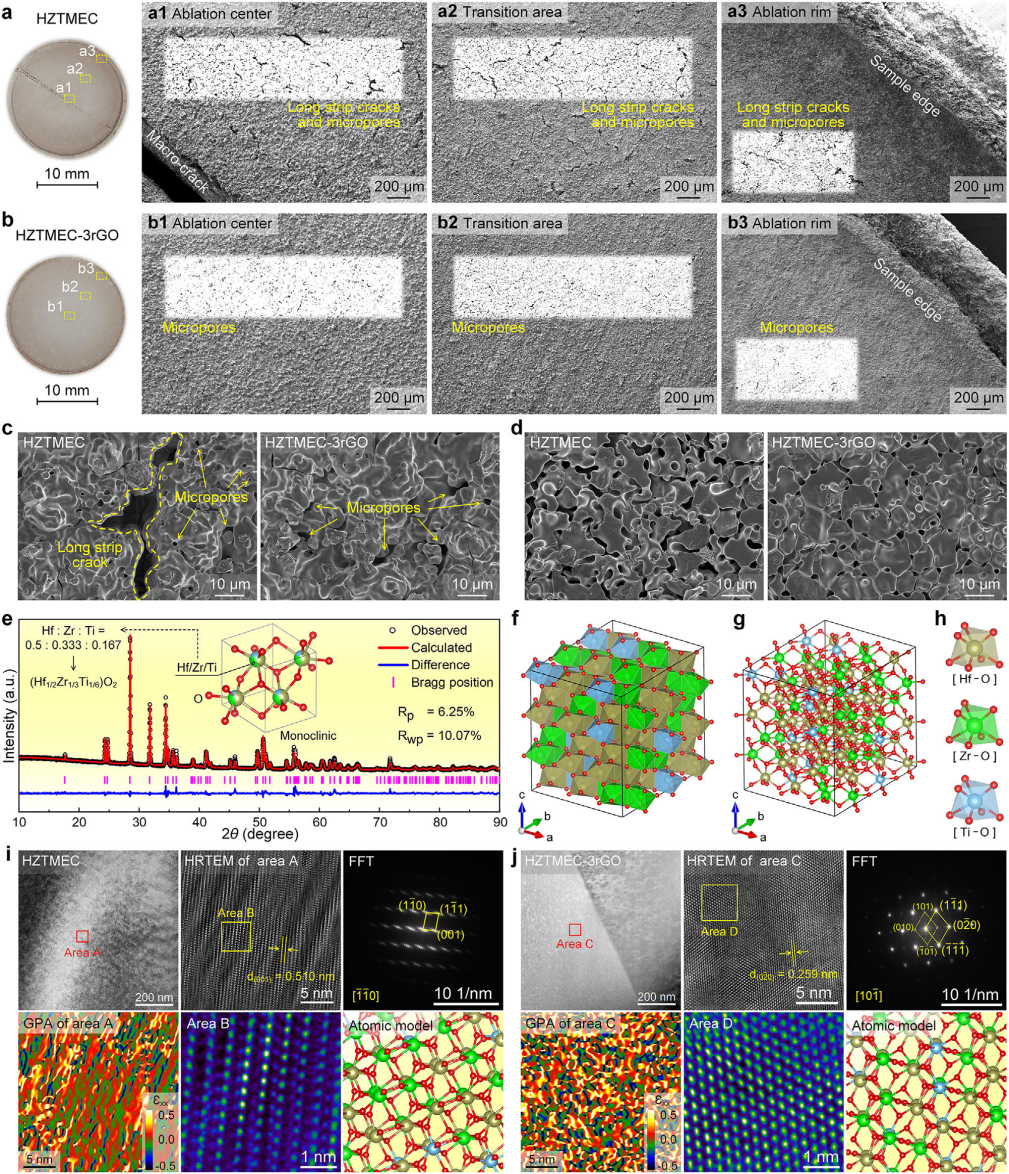
Analysis of the oxide layer after ablation. (a) Photo of HZTMEC after ablation: (a1−a3) show the SEM images of the HZTMEC oxide layer at the ablation center, transition area, and ablation rim, respectively. The rectangular areas have been contrast‐enhanced to highlight cracks and pores. (b) Photo of HZTMEC‐3rGO after ablation: (b1−b3) show the SEM images of the HZTMEC‐3rGO oxide layer at the ablation center, transition area, and ablation rim, respectively. (c) Comparison of the microstructure at the ablation center. (d) Comparison of the microstructure at the ablation rim. (e) Rietveld refinement of the XRD pattern of the HZTMEC‐3rGO oxide layer; the inset shows the corresponding unit cell structure diagram, which presents the crystal structure of the monoclinic phase (Hf_1/2_Zr_1/3_Ti_1/6_)O_2_. (f−h) The polyhedral structure, chemical bond structure of the monoclinic phase (Hf_1/2_Zr_1/3_Ti_1/6_)O_2_, and coordination models of Hf‐O, Zr‐O, and Ti‐O. (i) Phase and strain analysis of the HZTMEC oxide layer, followed by the HAADF image, the HRTEM image of area A, the FFT pattern, the GPA of area A, the enlarged image of area B in the HRTEM image, and the corresponding atomic model. (j) Phase and strain analysis of the HZTMEC‐3rGO oxide layer, followed by the HAADF image, the HRTEM image of area C, the FFT pattern, the GPA of area C, the enlarged image of area D in the HRTEM image, and its corresponding atomic model.

The ablation mechanisms can be analyzed by examining the oxide layer, as shown in Figure 5. Figure 5a,b present photographs of the oxide layers of HZTMEC and HZTMEC‐3rGO, respectively. The corresponding morphological features of the ablation center, transition area, and rim area are shown in Figure 5a1–a3 and Figure 5b1–b3, respectively. To highlight the cracks and pores, we applied contrast enhancement to typical areas, as indicated by the rectangular black and white regions. Specifically, two types of cracks were observed in the ablation surface of HZTMEC, i.e., the macro‐cracks and numerous long strip cracks with lengths of approximately 100–200 µm. The straight morphology of the macrocracks suggests a brittle, intergranular fracture mode. By contrast, long strip‐like cracks show a flexural path, propagating along weak regions. During ablation, high temperature and oxidation preferentially weaken ceramic grain boundaries, and thus these cracks mainly follow the boundaries. In contrast, the ablated surface of HZTMEC‐3rGO is devoid of cracks, featuring only sporadic micropores within an otherwise effective oxide‐protective layer.

Figure 5c,d present the micromorphology of the ablation center and rim under high magnification. As shown in Figure 5c, the main morphology of the ablation centers of both samples resembled a hilly terrain. Among these, HZTMEC exhibits both micropores and long strip cracks, while HZTMEC‐3rGO only has micropores and is relatively dense. Additionally, the morphologies of the ablation rims of both samples were relatively flat (Figure 5d). HZTMEC shows a looser structure with larger pores, while HZTMEC‐3rGO exhibits a denser structure with smaller pores. Overall, the results in Figure 5c,d are consistent with those in Figure 5a,b. Figure S12 presents cross‐sectional morphologies of the oxide layers. In HZTMEC, distinct transverse cracks are visible, and the oxide thickness on the ablation surface (598.73 µm) is comparable to that on the backside (551.64 µm). By contrast, HZTMEC‐3rGO shows a flatter, denser cross‐section with only minor cracks. Its ablation‐surface oxide thickness (570.01 µm) is similar to HZTMEC, whereas the backside oxide is much thinner (246.05 µm). These results indicate that the intact oxide layer in HZTMEC‐3rGO provides effective protection.

In addition to the structure of the oxide layer, the thermogravimetry‐differential scanning calorimetry (TG‐DSC) results in Figure S13 further reveal the oxidation behavior of the samples. For HZTMEC, the oxidation reaction was concentrated at peak P1 (corresponding to 992.54°C, exothermic 864.17 J/g), with a weight gain of 16.83%. In the case of HZTMEC‐3rGO, the oxidation reaction occurred in two distinct stages (P4 and P5). Peak P4 corresponds to 925.15°C (exothermic 3431.63 J/g), while peak P5 corresponds to 1228.68°C (exothermic 1269.50 J/g), with an overall weight gain of 15.36%. The smaller weight gain of HZTMEC‐3rGO compared to that of HZTMEC demonstrated the loss of rGO owing to oxidation. Therefore, P4 corresponds to the simultaneous oxidation of rGO and HZTMEC, indicating that the addition of rGO reduced the initial oxidation temperature and increased the heat release. P5 corresponds to the further oxidation of the inner layer of HZTMEC‐3rGO after the depletion of the surface rGO. At this stage, the micropores left by the oxidation of the surface rGO provided diffusion pathways for oxygen, thereby prolonging the oxidation process.

Considering the thermal shock and oxidation reactions during ablation, it is reasonable to speculate that the cracks in the ablation oxide layer of the HZTMEC are directly related to the thermal stress caused by severe thermal shock and the transformation stress caused by volume expansion during the oxidation reaction. However, HZTMEC‐3rGO is also inevitably affected by the above two types of stress but does not exhibit significant cracking on the surface or cross‐section of the oxide layer, which indicates the existence of a stress dissipation mechanism within its oxide layer. Therefore, it is necessary to analyze the oxide layer in terms of the phase, strain, and stress.

Figure S14 presents the XRD patterns of the oxide layers of HZTMEC, HZTMEC‐3rGO, and HZTMEC‐5rGO, all of which exhibited single‐phase characteristic peaks similar to those of monoclinic‐phase HfO_2_ (PDF#74‐1506). Figure S15 shows high‐angle annular dark field (HAADF) images and corresponding EDS maps of the oxide layers of HZTMEC and HZTMEC‐3rGO, indicating that Hf, Zr, Ti, and O were uniformly distributed without obvious segregation. A combination of Figures S14 and S15 demonstrates that the oxide layers are monoclinic (HfZrTi)O_2_. The Rietveld results for the HZTMEC‐3rGO oxide layer are shown in Figure 5e, and the specific crystal structure parameters are listed in Table S2. The refinement indices, R_p_ = 6.25% and R_wp_ = 10.07%, indicate that the refinement results are within a reliable range. From the refinement results, the space group of the monoclinic phase (HfZrTi)O_2_ was *P121/c1*, with an Hf:Zr: Ti ratio of 0.5:0.333:0.167, indicating that the atomic composition ratio was well preserved. The corresponding unit cell structure is shown in the inset of Figure 5e. Therefore, the lattice model of (HfZrTi)O_2_ can be considered as Zr and Ti atoms replacing Hf atoms in the HfO_2_ monoclinic phase. Furthermore, the overall models in Figure 5f,g are composed of Hf─O, Zr─O, and Ti─O coordination models, where each Hf/Zr/Ti atom is coordinated to seven O atoms. Therefore, the Hf─O, Zr─O, and Ti─O coordination models formed distorted octahedra (Figure 5h). In combination with Table S2, the unit cell volume of the monoclinic phase (HfZrTi)O_2_ is 137.964 Å^3^, which is significantly larger than that of HfZrTiC before ablation (98.27 Å^3^, Table S1), confirming the speculation that the oxidation reaction causes volume expansion, which is the primary factor contributing to the transformation stress.

In addition, severe thermal shock during ablation can generate thermal stress, which requires further strain analysis. Figure 5i shows the phase and strain analyses of HZTMEC, including HAADF, HRTEM of area A, FFT, GPA, an enlarged view of the HRTEM of area B, as well as the corresponding atomic model. After calibration, the low‐index diffraction spots in the fast Fourier transform (FFT) pattern in Figure 5i correspond to the $\left(10\bar{1}\right)$, $\left(11\bar{1}\right)$, and (001) crystal planes with the zone axis being $\left[\bar{1}\bar{1}0\right]$. The lattice fringe spacing of the (001) plane was 0.510 nm, as shown in the HRTEM image in Figure 5i. Notably, the HRTEM image of area A in Figure 5i exhibits an obvious Moiré pattern. After ruling out the factors related to the sample preparation methods and equipment, the formation of the Moiré pattern can be attributed to the misalignment of atomic columns, which is associated with the nonuniform deformation of the sample, possibly caused by factors such as ablation or fracture. Furthermore, GPA in area A shows atomic strain with an anomalous orientation, supporting the presence of nonuniform deformation. The enlarged HRTEM image of area B reveals misaligned atomic columns. Using the HRTEM image (Figure 5i), FFT pattern, and atomic model (Figure 5g), we reconstructed the arrangement in area B, confirming the column misalignment in the atomic model.

Figure 5j shows the phase and strain analyses of HZTMEC‐3rGO, including HAADF and HRTEM images of area C, FFT, GPA, an enlarged view of HRTEM in area D, and the corresponding atomic models. The HAADF image in Figure 5j shows a clear grain boundary. Because the oxide layer was single‐phase, area C was selected for further analysis. After measurement and calibration, the three main diffraction spots in the FFT pattern in Figure 5j correspond to the $\left(11\bar{1}\right)$, $\left(00\bar{2}\right)$, and $\left(\bar{1}\bar{1}\bar{1}\right)$ crystal planes, with the zone axis as $\left[10\bar{1}\right]$. The lattice fringe spacing of the $\left(00\bar{2}\right)$ crystal plane is 0.259 nm, as shown in the HRTEM image in Figure 5j. In the GPA, the atomic strain distribution in region C of Figure 5j was uniform, which differed significantly from that in Figure 5i. Furthermore, the enlarged image of area D shows a regular atomic arrangement without a Moiré pattern or misalignment of the atomic columns. The atomic arrangement in region D was reconstructed by combining the HRTEM image and corresponding FFT pattern (Figure 5j) with reference to the structural model in Figure 5g.

The distinct oxide‐layer morphology and lattice structure in HZTMEC‐3rGO, compared to HZTMEC, demonstrate that rGO doping effectively modifies the strain distribution within the layer. To verify this, we analyzed the XRD data using the Williamson–Hall method (Figure S16). Among them, HZTMEC exhibits negative strain (−0.087), while HZTMEC‐3rGO exhibits positive strain (0.051). Additionally, strip‐shaped micropores were discovered on the oxide layer surface of HZTMEC‐3rGO (Figure S17), with dimensions (approximately 50 µm) similar to those of rGO in Figure 2a, suggesting they originate from voids left by oxidation and volatilization of rGO. Strip‐shaped micropores across the ablation surface (Figure 5b) provide buffer space for volumetric and thermal expansion, dispersing transformation and thermal stresses. This yields more uniform atomic strain (GPA, Figure 5j) and increased positive strain (Figure S16b), helping to prevent brittle fracture. For the HZTMEC, there was no dispersion of the transformation stress and thermal stress. This implies that the volume expansion caused by oxidation and thermal expansion caused by thermal shock squeeze the crystal lattice, leading to the accumulation of transformation and thermal stresses. Eventually, stress concentration occurs at the ablation center, the region most severely affected by plasma flame thermal shock. This leads to the formation of brittle cracks that penetrate the center, accompanied by numerous long strip cracks (100–200 µm) within the corresponding oxide layer (Figure 5a). Therefore, the atomic strain of the HZTMEC after ablation exhibited an abnormal orientation (GPA in Figure 5i), which is consistent with the negative strain results in Figure S16a.

To support the theory that strip‐shaped micropores possess a stress‐dissipation function, Figure S18 presents the finite element simulation results for HZTMEC and HZTMEC‐3rGO during the ablation heating period. Figure S18a shows a 3D model of the samples, where HZTMEC‐3rGO exhibits strip‐shaped micropore structures distributed on the surface. Figure S18b illustrates the temperature distribution of the samples, revealing a high temperature at the ablation center that rapidly increases from 1400°C to 2400°C within approximately 30 s, consistent with the actual ablation curve and verifying the rationality of the simulation. Figure S18c presents the equivalent stress distribution. Maximum stress concentrated at the ablation center, directly exposed to the heat flux, and expanded outward as the temperature rose. Compared to HZTMEC, HZTMEC‐3rGO showed markedly lower equivalent stress, especially at 10 and 20 s. The difference was the most obvious at 20 s; the equivalent stress at the center of HZTMEC was approximately 500 MPa, whereas that of HZTMEC‐3rGO was only approximately 425 MPa. Additionally, Figure S18d illustrates the distribution of the first principal stress on the surface and cross‐section of the samples. Compressive stress was observed at the sample center, while tensile stress was present at the edges. This is consistent with experiments in which the sample center underwent thermal shock, while the edges experienced thermal expansion. Comparisons revealed that the tensile stress at the edges of HZTMEC‐3rGO was significantly lower than that of HZTMEC, with the difference being apparent at 30 and 40 s. The most obvious difference occurred at 40 s: the tensile stress at the edges of HZTMEC approached 200 MPa, whereas that of HZTMEC‐3rGO was approximately 75 MPa. Overall, both the equivalent stress and first‐principles stress results support the argument that strip‐shaped micropore structures function in stress dissipation.

**FIGURE 6:**
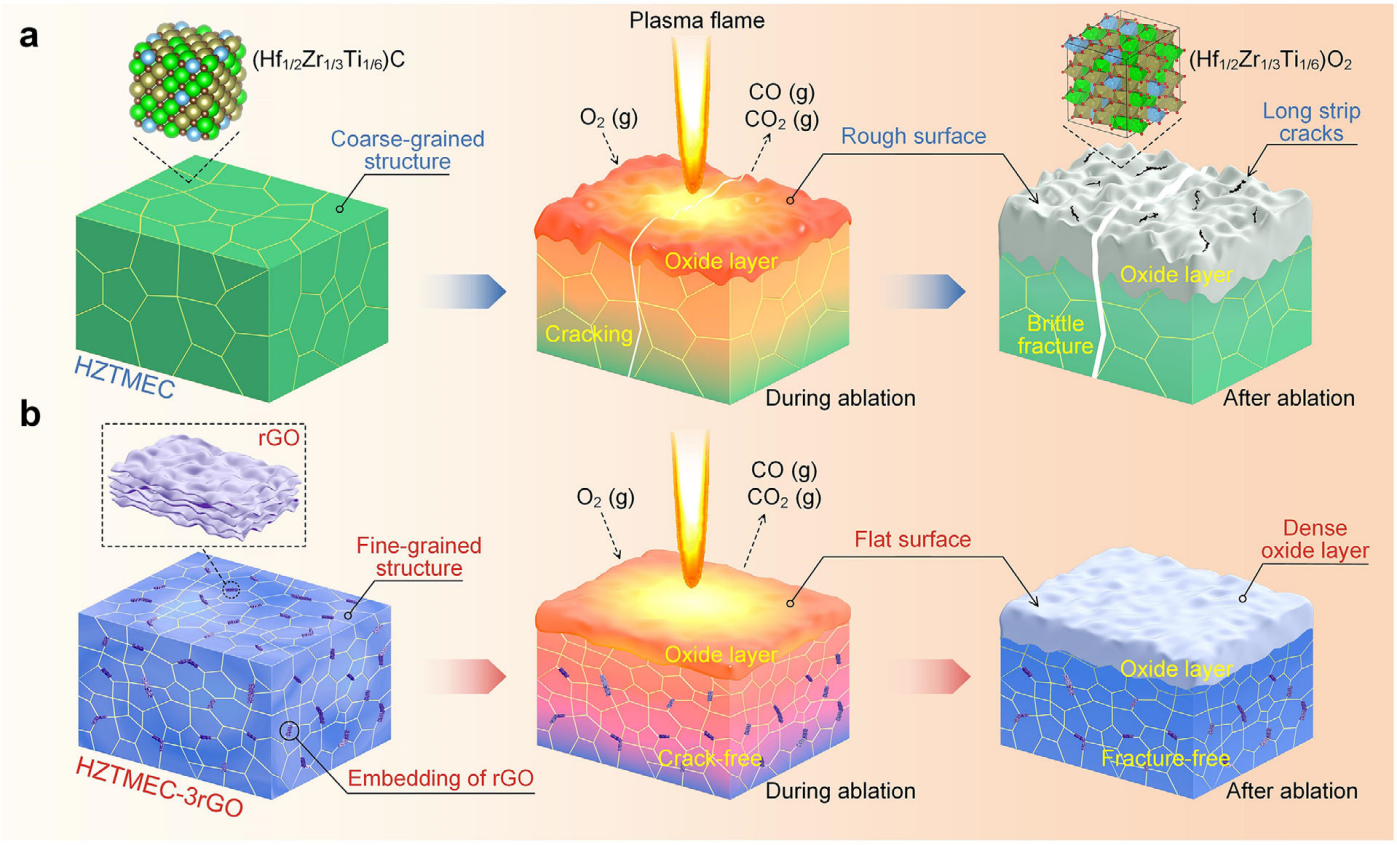
Schematic of ablation mechanism. (a) Ablation evolution process of HZTMEC, in sequence: the coarse‐grained structure of carbide ceramics before ablation; thermal shock, oxidation, and cracking during ablation; and the rough, cracked oxide layer and brittle fracture after ablation. (b) Ablation evolution process of HZTMEC‐3rGO, in sequence: the fine grain structure and rGO doping before ablation; thermal shock and oxidation during ablation; and the relatively flat and dense oxide layer after ablation, in which no fracture occurred.

Based on the aforementioned results, Figure 6 shows the ablation mechanisms of HZTMEC and HZTMEC‐3rGO, highlighting the primary differences between the two materials during the ablation evolution process. Before ablation, the HZTMEC exhibited relatively coarse grain sizes, whereas the addition of rGO inhibited grain growth, resulting in finer grains in HZTMEC‐3rGO. The addition of rGO modified the strain of HZTMEC‐3rGO at the nano‐ and micrometer scales, resulting in increased atomic and grain strains, which increased the dislocation density and hindered dislocation movement, thereby improving the *K_IC_* of HZTMEC‐3rGO. When subjected to ablation temperatures up to 2400°C, HZTMEC developed significant cracking, with its oxide layer becoming both cracked and relatively rough. By contrast, the HZTMEC‐3rGO remained intact. The oxide layer was flatter and denser, providing more effective protection, as evidenced by the significant reductions in MAR and LAR.

A second mechanism also contributes. During ablation, rGO in HZTMEC‐3rGO oxidizes and volatilizes, leaving strip‐shaped micropores in the oxide layer. These pores disperse transformation and thermal stresses, promoting a more uniform strain field and preventing stress concentration and brittle fracture. By contrast, in undoped HZTMEC, oxidation and thermal shock drive large volumetric and thermal expansion, causing stress buildup; the resulting atomic strain shows anomalous orientations, leading to stress concentration at the ablation center and brittle cracks that penetrate this region.

## 3 Conclusions

In summary, we have developed a novel thermal protection ceramic, HZTMEC‐3rGO, which demonstrates great potential for long‐term service at temperatures up to 2400°C, and its *K_IC_* reaches 7.514 MPa·m^1/2^. Compared with HZTMEC, the MAR and LAR of HZTMEC‐3rGO were significantly reduced by 22.4% and 43.9%, respectively. Moreover, the resulting protective oxide layer was flatter and denser, maintaining good structural integrity. This breakthrough originated from the hierarchical strain modification induced by rGO doping, which involved an inhibition mechanism for crack propagation at the microscale and a stress‐dissipation mechanism at the mesoscale. The former manifests as an increase in atomic stress and the expansion of high‐strain regions. The latter arises from the rGO acting as a sacrificial phase during ablation, where the strip‐shaped micropores left by oxidation and volatilization effectively dissipate stress, thereby helping to maintain the structural stability of the oxide layer. Thus, HZTMEC‐3rGO avoids brittle fracture in thermal‐mechanical‐oxidative coupled ablation environments.

The proposed hierarchical strain modification strategy provides a new paradigm for balancing ultrahigh‐temperature ablation resistance and structural stability. This design can be extended to other ablation‐resistant ceramic systems, thereby promoting the development of thermal protection systems for aircraft. Future research should focus on the type and spatial distribution of doped phases to optimize the composition and structure, achieving breakthroughs in higher temperature ranges.

## 4 Methods

### 4.1 The First‐Principles Calculation

First‐principles calculations were performed based on density functional theory (DFT) and the local density approximation (LDA) [49]. Atomic models and a special quasi‐random structure (SQS) were constructed using VESTA, Materials Studio, and VASP. Calculations were performed using the DFT provided by the VASP code, with the Perdew–Burke–Ernzerhof (PBE) functional under the generalized gradient approximation (GGA) used for solving electron exchange interactions [50, 51]. The (100) crystal plane was the primary exposed crystal face determined by calculating the surface formation energy (Figure S19). The main parameters for calculation and modeling are listed in Table S3 [52]. The core formulae for calculating the surface formation energy *E*_surf_ and the oxygen adsorption energy *E*_ads_ are shown in Equations (5) and (6), respectively [35].

(5) $$E_{\mathrm{surf}}=\frac{E_{\mathrm{slab}}-nE_{\mathrm{bulk}}}{2A}$$

(6) $$E_{\mathrm{ads}}=E_{\mathrm{slab}+O}-E_{\mathrm{slab}}-E_{O}$$

where *E*_slab_ and *E*_slab+O_ are the total energies of the surface models with and without O atom adsorption, respectively, *n* is the ratio of atoms in the surface model to those in the crystal cell model, *E*_bulk_ is the total energy of the crystal cell model, *A* is the cross‐sectional area of the surface model, and *E*_O_ is the energy of a single O atom.

### 4.2 Material Synthesis

The original powders include HfC, ZrC, TiC, and GO, where the purity of the carbide ceramic powders is >99%, with particle sizes of approximately 2–5 µm, and the GO sheet diameter is 11–15 µm. The molar ratio of the HfC, ZrC, and TiC powders was 3:2:1. The original samples were circular discs with a diameter of 45 mm and a thickness of 5 mm. GO was added at 1, 3, and 5 vol% based on the sample dimensions. The original powders were mixed 10 times using a biaxial mixer (Speedmixer DAC 600) at a mixing speed of 1200 rpm and a mixing time of 600 s. Compared with liquid‐phase mixing, biaxial solid‐phase mixing sacrifices some dispersibility but, via high‐speed biaxial rotation, imparts greater kinetic energy and centrifugal force; it also eliminates liquid‐medium stripping steps, improving efficiency and preserving powder purity. A graphite mold with a filling diameter of 45 mm and a hot‐press sintering furnace (ZH‐40‐21Y) were used to complete sintering and synthesis (maintain a pressure of 30 MPa at 2100°C for 1 h). Taking advantage of the properties of GO, which can be easily reduced to rGO during heat treatment, the sintering process of ceramics is combined with GO reduction. The solid‐solution sintering reaction of the HZTMEC and the in situ reduction of rGO were completed simultaneously, ultimately achieving the addition of rGO to the HZTMEC.

### 4.3 Microstructure Characterization and Testing

The phase structure of the samples was analyzed using XRD (Bruker‐D8 ADVANCE) with Cu Kα irradiation. The XRD patterns were refined using TOPAS software and the Rietveld method [53]. Strain analysis of the XRD patterns was performed using MDI Jade 9 software and the Williamson–Hall method [54]. The microstructure and elemental mapping of the samples were characterized by scanning electron microscopy (SEM, Sigma 300) and energy‐dispersive X‐ray spectroscopy (EDS, Ultimax), respectively. The EBSD was mounted on the SEM to obtain the IPF and KAM of the samples. An FIB (DB500) was used to prepare the samples for TEM characterization, and HRTEM (FEI Talos F200) was used to characterize the nanostructures and elemental distributions of the samples. Further analysis of the HRTEM images was performed using the DigitalMicrograph software to obtain the GPA, FFT, and IFFT patterns [55, 56].

### 4.4 Evaluation of Basic Properties

Densities were measured by Archimedes’ method. Thermal conductivity was obtained on a comprehensive physical property system (Quantum Design PPMS‐9). Post‐ablation surface roughness was measured with an optical profilometer (Mahr MarSurf LD130). Fracture toughness was determined by the SENB method on a microcomputer‐controlled universal tester (C45.105), following ASTM E1820‐06 [57]. The core formula for calculating *K_IC_* is given by Equations (7) and (8).

(7) $$K_{IC-SENB}=\frac{FL}{BW^{\frac{3}{2}}}f\left(\frac{a}{W}\right)$$

(8) $$\begin{array}{ccc} & & f\left(\frac{a}{W}\right)\\ & & \;=\frac{3{\left(\frac{a}{W}\right)}^{\frac{1}{2}}\left[1.99-\frac{a}{W}\left(1-\frac{a}{W}\right)\right]\times \left[2.15-3.93\frac{a}{W}+2.7{\left(\frac{a}{W}\right)}^{2}\right]}{2\left(1+2\frac{a}{W}\right){\left(1-\frac{a}{W}\right)}^{\frac{3}{2}}} \end{array}$$

where *F* is the maximum load in the SENB test, *L* is the support span, *B* is the sample thickness, *W* is the sample width, and *a* is the notch depth.

### 4.5 Ablation Testing

Plasma flame ablation tests were conducted on the samples. Plasma was generated by the ionization and excitation of argon and nitrogen gases. Ar served as the primary gas (with a real‐time flow rate of 60 L/min), whereas nitrogen served as an auxiliary gas (with a real‐time flow rate of 14 L/min). During ablation, the real‐time current was approximately 600 A, the working voltage was approximately 75 V, the device power was controlled at 45 kW, the ablation time was 240 s, and the ablation distance was 60 mm. A multifunctional two‐color infrared thermometer (METIS M322, temperature measurement range 1000–3300°C) equipped with an integrated lens assembly was used to observe the ablation process and record the surface temperature. An infrared thermal imager (FLIR A3 series) was used to observe the ablation surfaces. MAR and LAR were calculated using Equations (9) and (10), respectively:

(9) $$MAR=\frac{m_{0}-m_{1}}{t}$$

(10) $$LAR=\frac{l_{0}-l_{1}}{t}$$

where *m*_0_ and *m*_1_ are the sample masses before and after ablation, respectively; *l*_0_ and *l*_1_ are the sample thicknesses before and after ablation, respectively; and *t* is the ablation time.

### 4.6 Finite Element Simulation

Finite‐element simulations (COMSOL Multiphysics) used settings matching the ablation experiment. The model dimensions matched the samples (20 mm diameter, 5 mm thickness). Strip‐shaped micropores—reflecting the oxide‐layer pores and rGO morphology—were simplified as strip‐like surface micro‐indentations distributed across the top surface. The model bottom was fixed. A circular surface heat source (6 mm diameter, equal to the plasma gun's inner diameter) was applied with a normal load to replicate the ablation environment. The simulation focused on the 1400–2400°C heating stage, where temperature and stress evolve prominently. In this regime, samples oxidize to a single phase, allowing the use of reference material properties (Table S4) [25, 58, 59].

### 4.7 Statistical Analysis

In data preprocessing, retain a maximum of four significant digits after the decimal point. The results are presented as the mean or mean ± standard deviation. Data obtained by manual counting, the sample size (n) can be obtained from the relevant figure or figure legend. For data obtained by software analysis, the outlier evaluation and n are both based on the default settings of the relevant software. Software used for statistical analysis includes Origin, AZtecCrystal, Jade, and ImageJ.

## Author Contributions

Pengfei He, Jiangbo Cheng, and Xiubing Liang conceived and designed the research and analysis. Junyi Xiao, Shujun Hu, and Jiangbo Cheng contributed to the data collection. Junyi Xiao, Lin Xue, and Liliang Shao performed the data analysis and wrote the paper with assistance from all authors. All authors contributed to the analysis of the results and the discussion in the manuscript.

## Funding

This work was supported by the National Natural Science Foundation of China (Grant No. 52375180) and the Fundamental Research Funds for the Central Universities (Grant Nos. B250205044, B250201219, and B240201072).

## Conflicts of Interest

The authors declare no conflict of interest.

## Supporting information




**Supporting File**: advs74041‐sup‐0001‐SuppMat.docx.

## Data Availability

The data that support the findings of this study are available from the corresponding author upon reasonable request.
