# Supplementary material for: Hierarchical Strain‐Modified Medium‐Entropy Carbide Ceramics Exhibit Exceptional Ablation Resistance up to 2400°C
Source: Adv Sci (Weinh). 2026 Jan 28;13(19):e18785. doi: 10.1002/advs.202518785 (PMC13045472; doi:10.1002/advs.202518785)
Supplement: Supplementary file 1 — Supporting File: advs74041‐sup‐0001‐SuppMat.docx. [file ADVS-13-e18785-s001.docx]

**Supplementary Information**

**Hierarchical Strain-Modified Medium-Entropy Carbide Ceramics Exhibit Exceptional Ablation Resistance up to 2400 °C**

*Junyi Xiao^a,b^, Pengfei He^b,^*, Lin Xue^a,^*, Shujun Hu^b^, Liliang Shao^a^, Chuan Sun^b^, Yunyun Ge^a^,* *Jiangbo Cheng^a,^*, Xiubing Liang^b,^**

*^a^ College of Materials Science and Engineering, Hohai University, Changzhou 213200, China*

*^b^ Defense Innovation Institute, Academy of Military Sciences, Beijing 100071, China*

**Corresponding authors:* [hepengfei93@163.com](mailto:hepengfei93@163.com) (Pengfei He); [linxue06@163.com](mailto:linxue06@163.com) (Lin Xue); [chengjiangbo@hotmail.com](mailto:chengjiangbo@hotmail.com) (Jiangbo Cheng); [liangxb_d@163.com](mailto:liangxb_d@163.com) (Xiubing Liang)

**This file includes:**

Figure S1: Evidence for the formation of rGO by oxygen desorption of GO after sintering.

Figure S2: Evidence of the atomic ratio of Hf-Zr-Ti in HZTMEC being 1/2 : 1/3 : 1/6.

Figure S3: Micro-morphology of HZTMEC-5rGO after grinding and polishing.

Figure S4: Evidence of rGO agglomeration.

Figure S5: Physical property test results for HZTMEC, HZTMEC-3rGO, and HZTMEC-5rGO.

Figure S6: The distribution of rGO in HZTMEC.

Figure S7: Dislocation and lattice distortion distribution in HZTMEC and HZTMEC-3rGO.

Figure S8: Ablation curve and photographs of HZTMEC-1rGO and HZTMEC-5rGO.

Figure S9: The load-displacement curves of SENB tests.

Figure S10: Atomic models for first-principles calculation.

Figure S11: Cubic spline interpolation based on *K_IC_* data of each sample.

Figure S12: Cross-sections of the oxide layers.

Figure S13: TG-DSC curves, showing the oxidation behavior of the samples.

Figure S14: The XRD patterns of the oxide layers.

Figure S15: HAADF and corresponding elemental mapping of the oxide layers.

Figure S16: Strain analysis results of the oxide layer based on the Debye-Scherrer formula.

Figure S17: Strip-shaped micropores on the oxide layer surface of HZTMEC-3rGO.

Figure S18: Finite element simulation results of HZTMEC and HZTMEC-3rGO during ablation.

Figure S19: Surface formation energy of low-index crystal faces of HZTMEC.

Table S1: Crystal structure parameters of HZTMEC from Rietveld refinement of XRD.

Table S2: Crystal structure parameters of the oxide layer of HZTMEC-3rGO.

Table S3: The main parameters of the first-principles calculation and modeling.

Table S4: Attribute parameters of material in finite element simulation.

**Supplementary Figures**

**
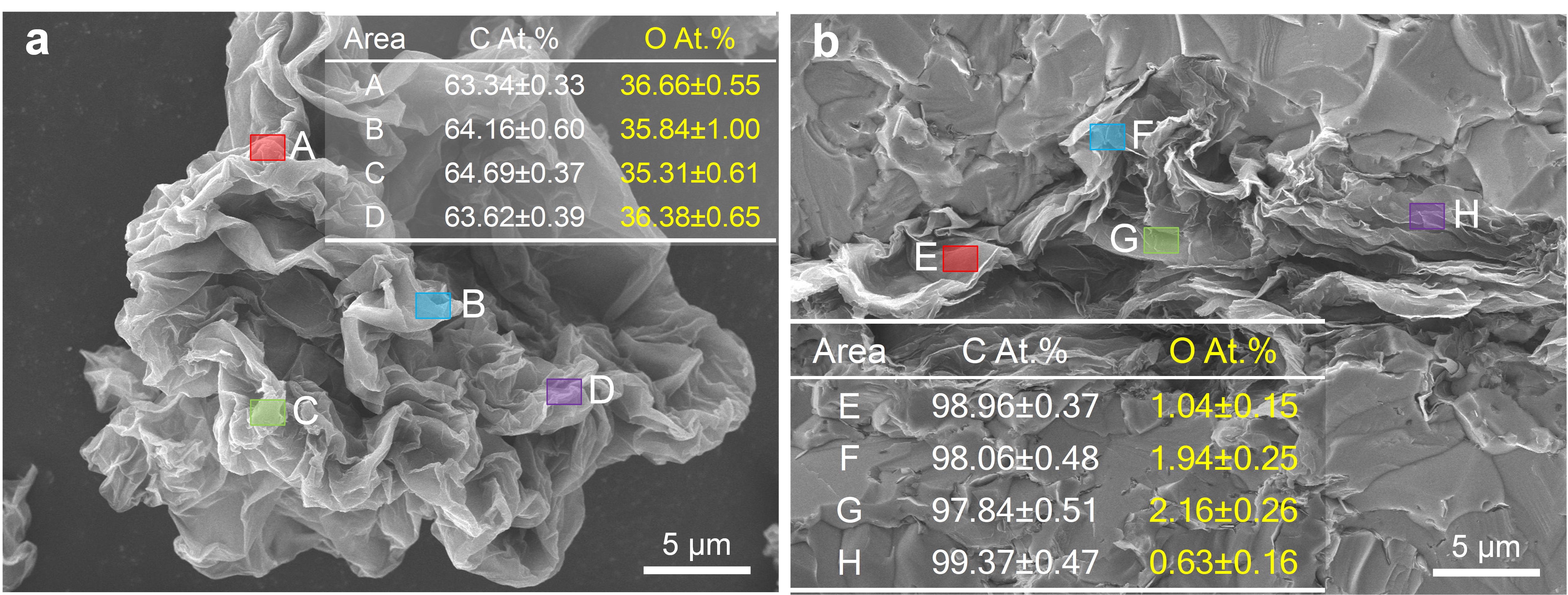
**

**Figure S1 Evidence for the formation of rGO by oxygen desorption of GO after sintering. a** Elemental content of GO before sintering. **b** Elemental content of rGO after sintering.


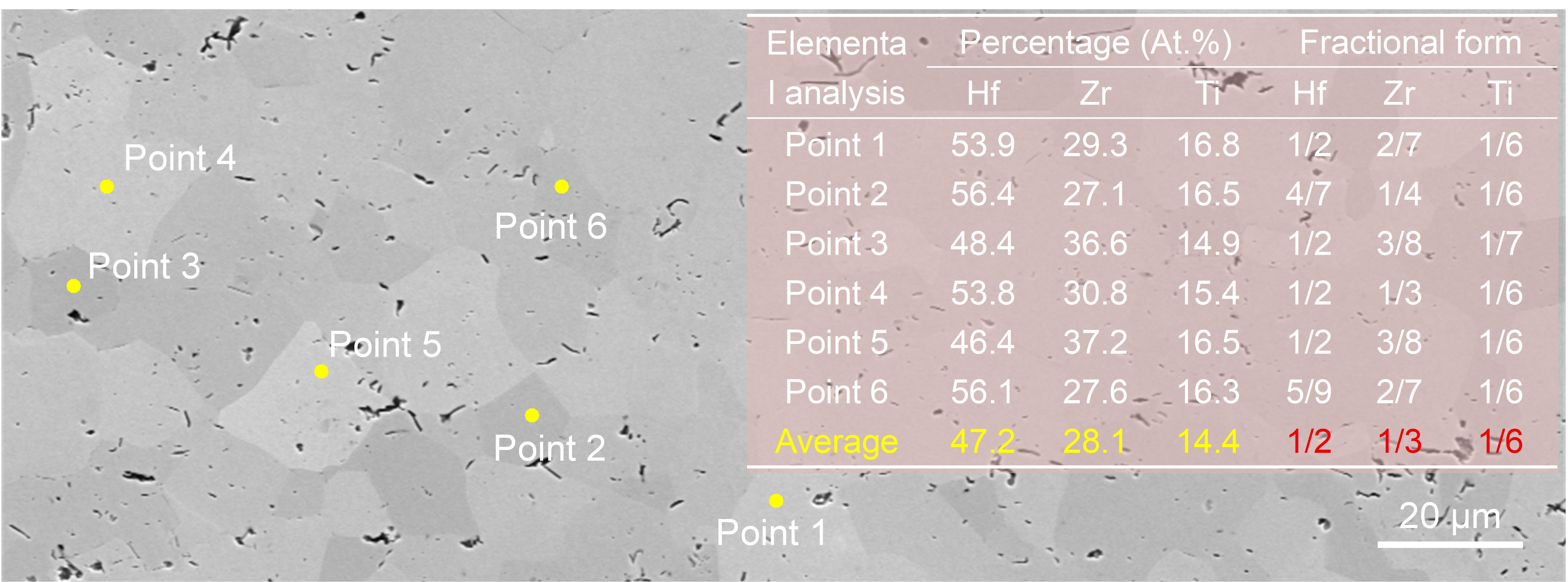


**Figure S2** **Evidence of the atomic ratio of Hf-Zr-Ti in HZTMEC being 1/2 : 1/3 : 1/6.**

**
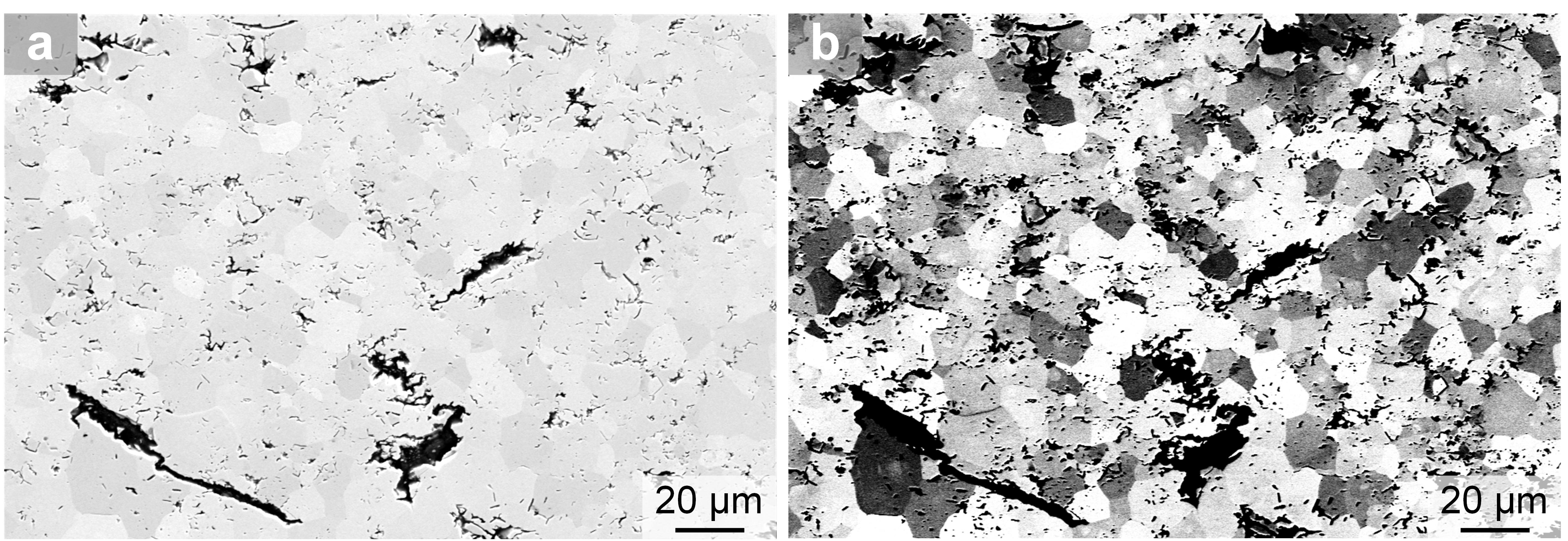
**

**Figure S3** **Micro-morphology of HZTMEC-5rGO after grinding and polishing. a** SEM imaging. **b** BSE imaging.

**
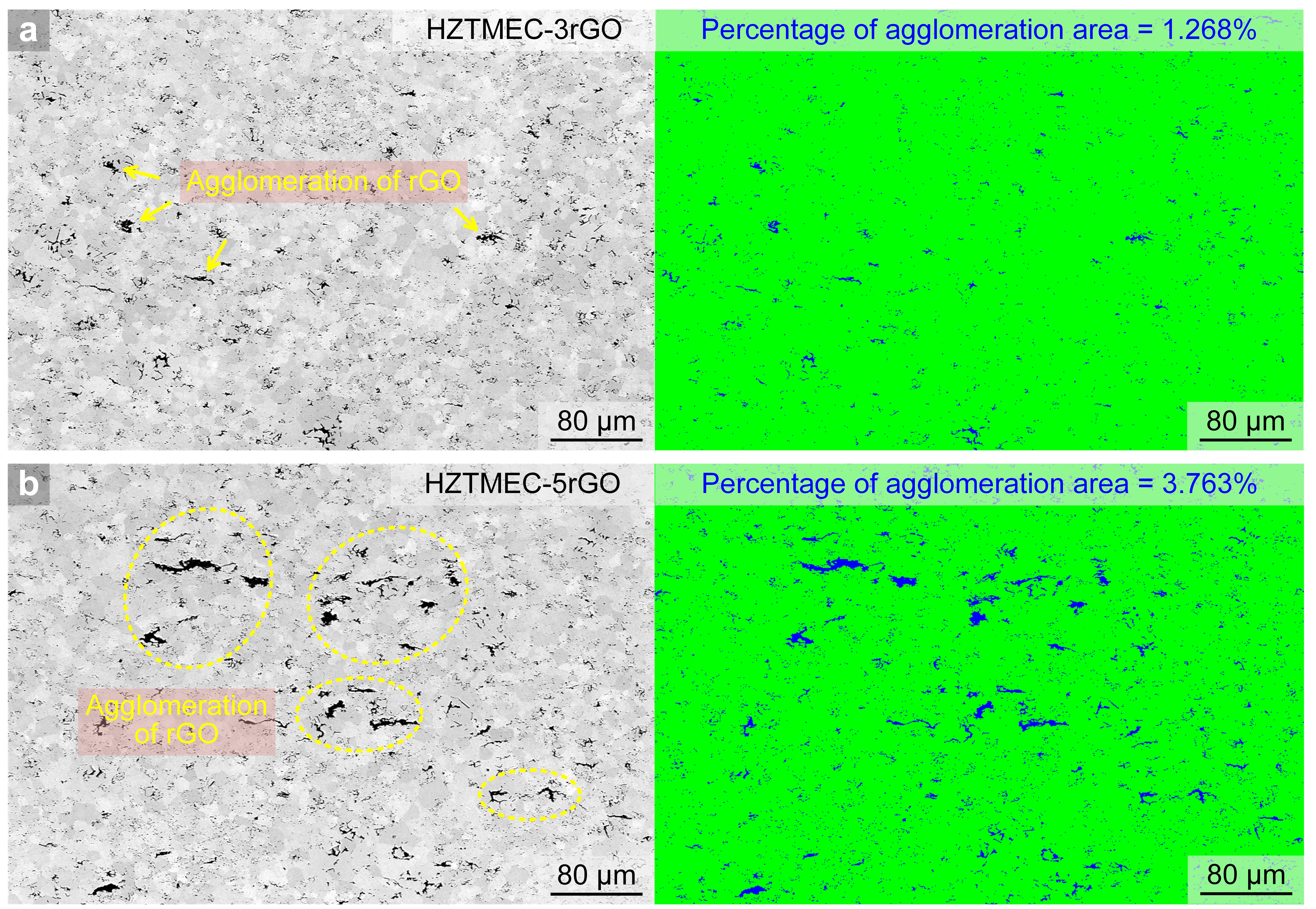
**

**Figure S4 Evidence of rGO agglomeration, with the percentage of corresponding agglomeration areas by pixel identification method using ImageJ software. a** HZTMEC-3rGO. **b** HZTMEC-5rGO.


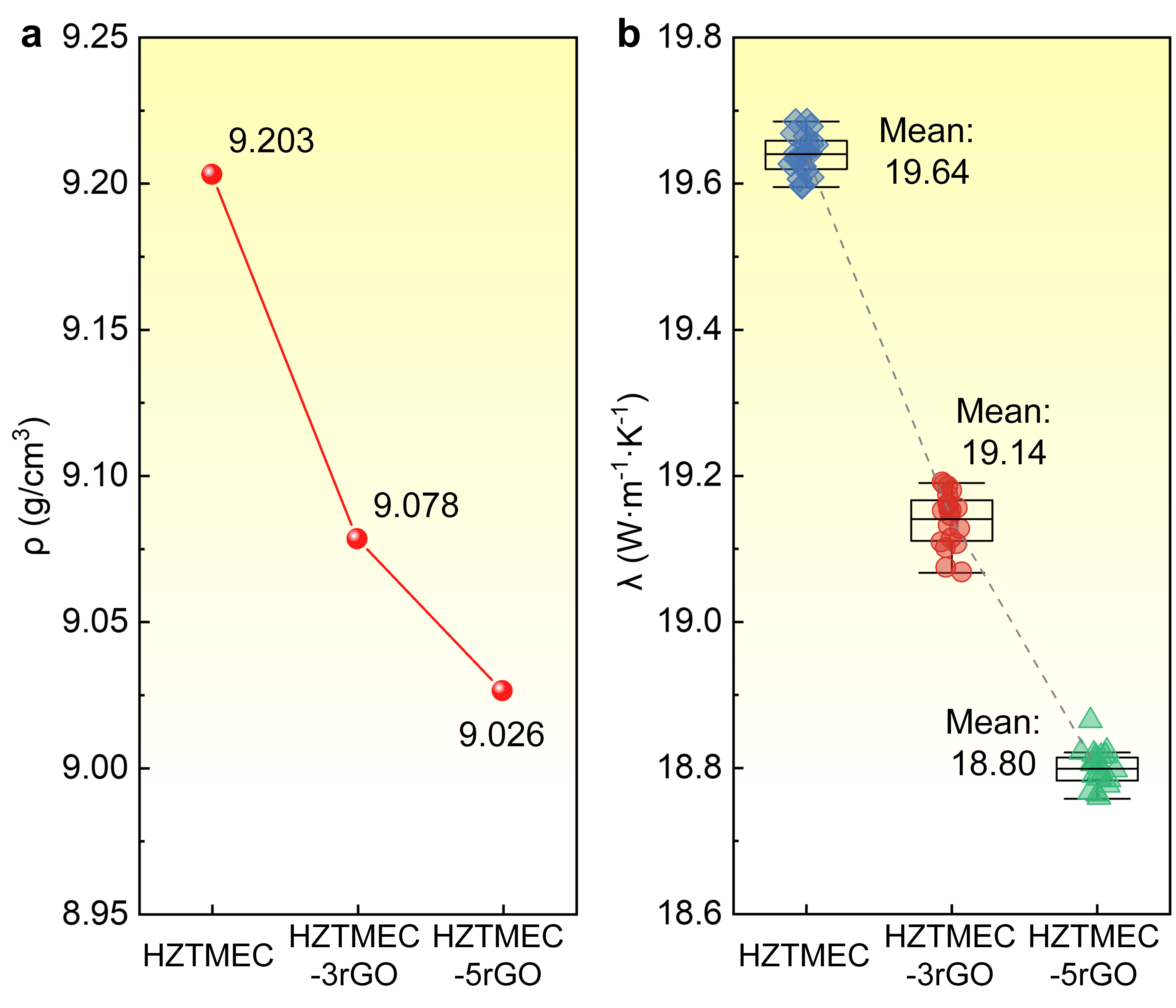


**Figure S5** **Physical property test results for HZTMEC, HZTMEC-3rGO, and HZTMEC-5rGO. a** Density. **b** Thermal conductivity (n = 20).


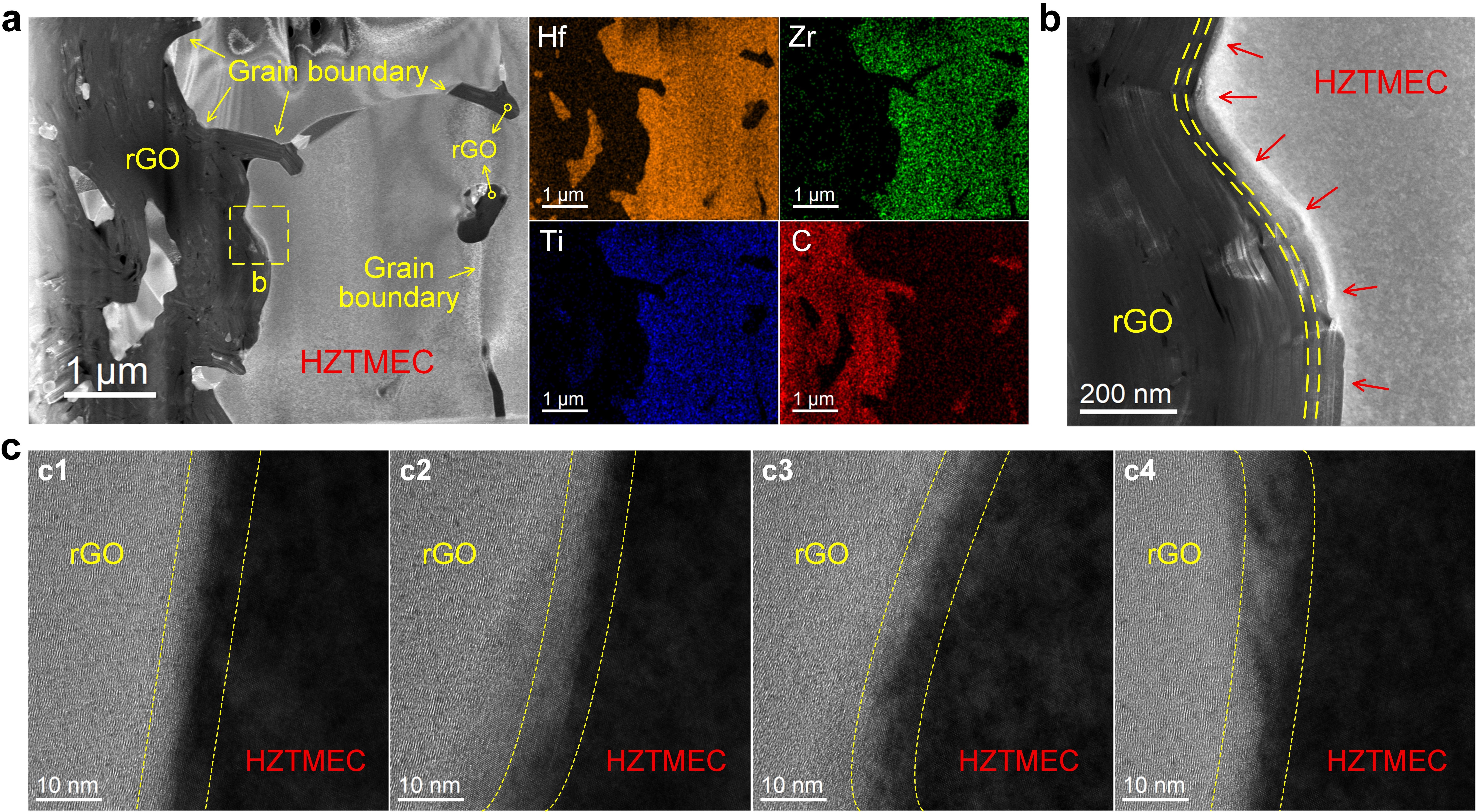


**Figure S6 The distribution of rGO in HZTMEC.** **a** The DF image and EDS maps of the complete sample. **b** The DF image of the interface between HZTMEC grains and rGO. **c** HRTEM images of the rGO/HZTMEC interface at several different locations.


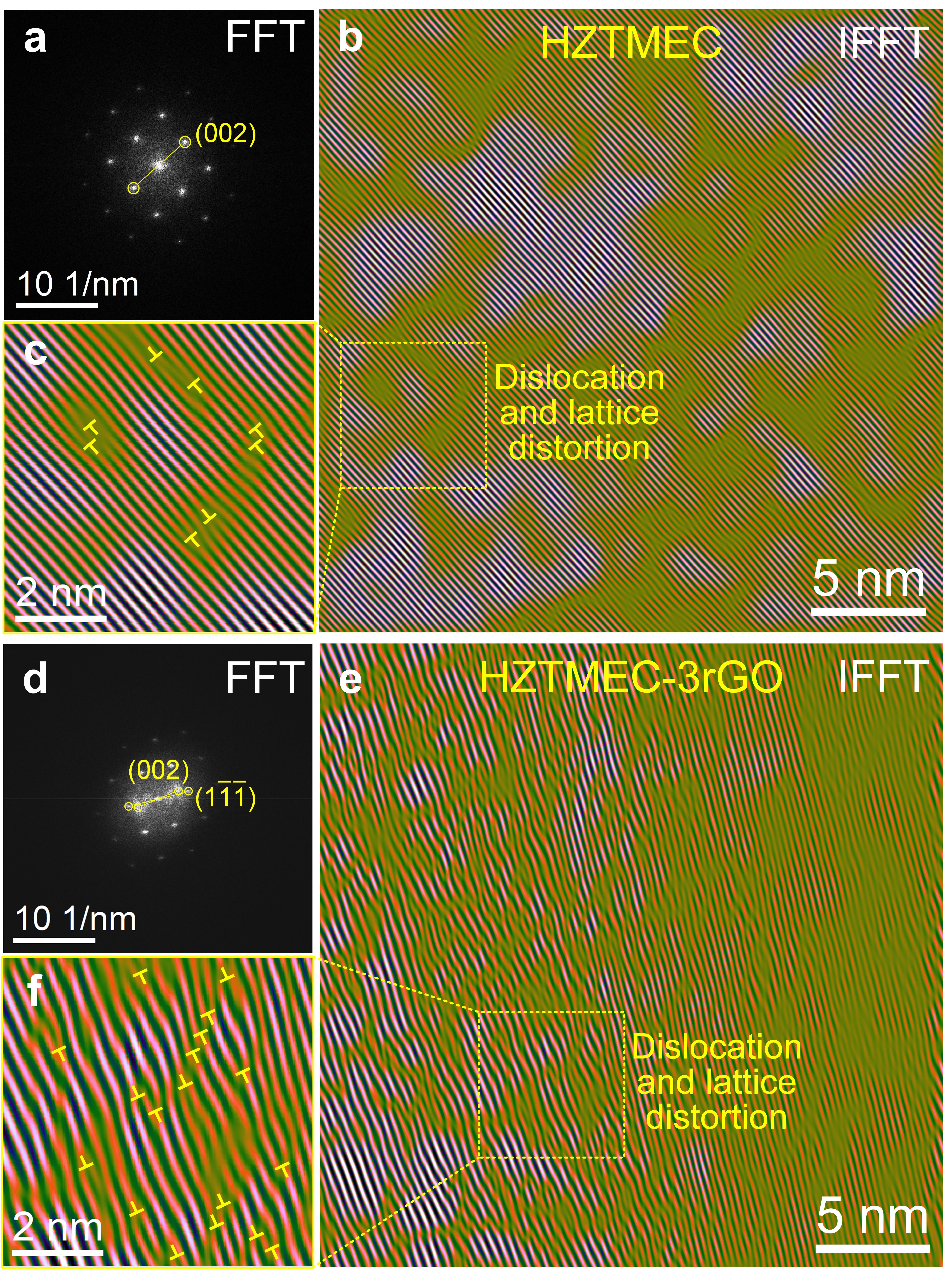


**Figure S7** **Dislocation and lattice distortion distribution in HZTMEC and HZTMEC-3rGO.** **a** FFT pattern of HZTMEC (based on Figure 3e). **b** IFFT pattern obtained from Figure a, where the pixel recognition and coloring functions of DigitalMicrograph software were used to highlight the dislocation and lattice distortion regions (green regions) and the regular lattice regions (light pink regions). **c** Partial enlarged detail of Figure b, marking the specific locations of dislocations. **d** FFT pattern of HZTMEC-3rGO (based on Figure 3f). **e** IFFT pattern obtained from Figure d. **f** Partial enlarged detail of Figure e, marking the specific locations of dislocations.


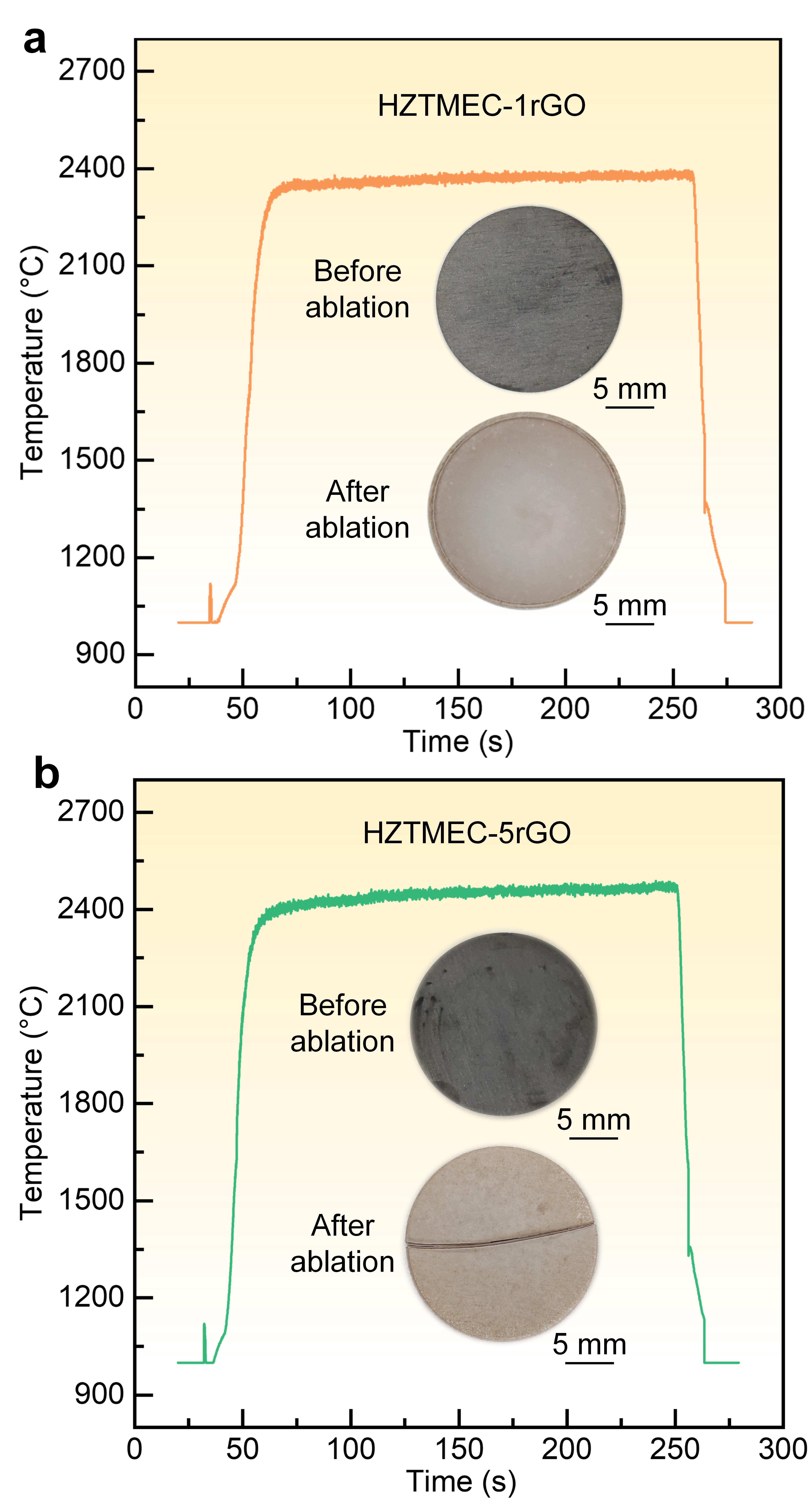


**Figure S8** **Ablation curve of other samples under the same testing conditions, with insets showing photographs before and after ablation. a** HZTMEC-1rGO. **b** HZTMEC-5rGO.


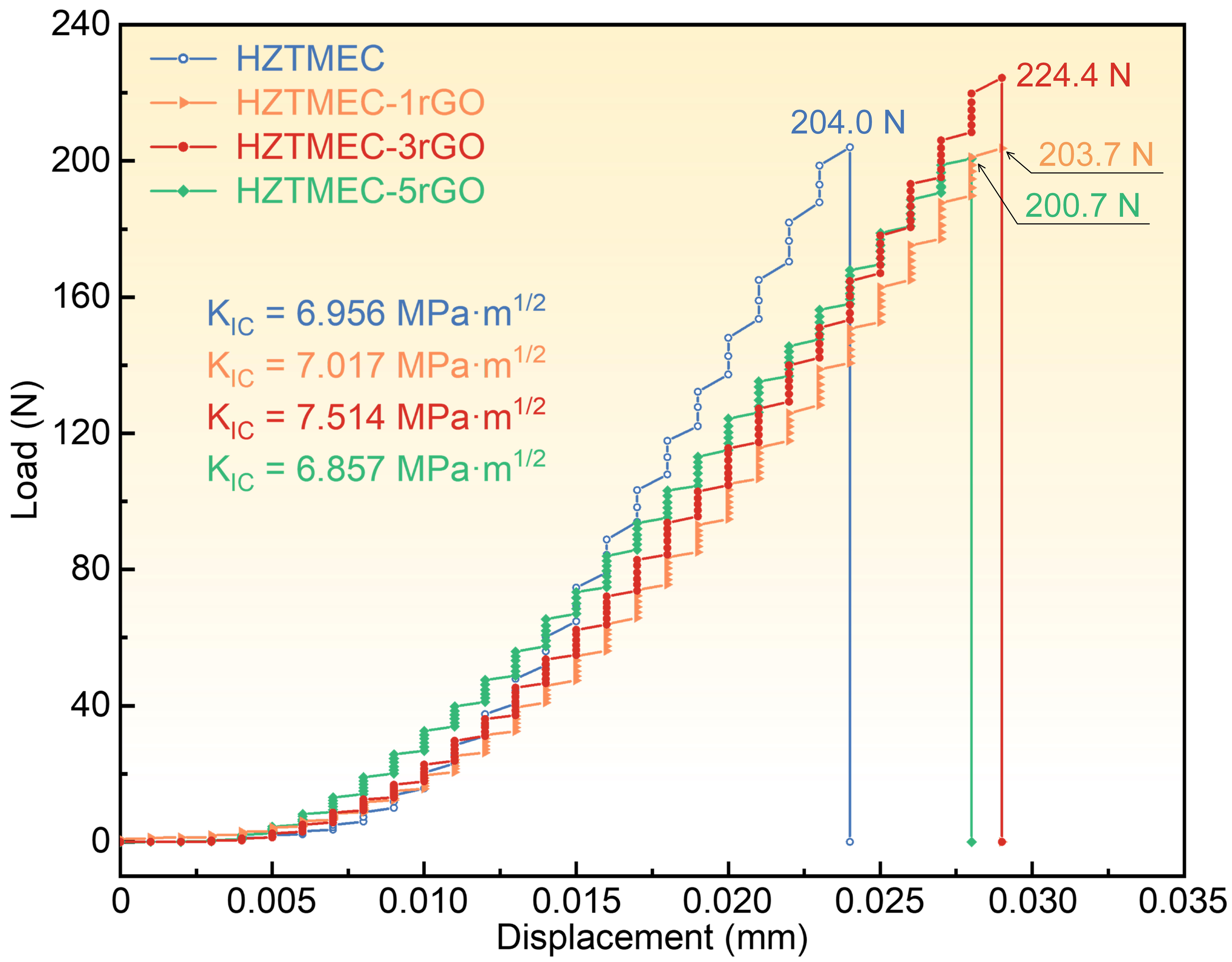


**Figure S9** **The load-displacement curves of SENB** **tests, including HZTMEC, HZTMEC-1rGO, HZTMEC-3rGO, and HZTMEC-5rGO.**

**

**

**Figure S10** **Atomic models for** **first-principles calculation. a** HZTMEC. **b** HZTMEC-3rGO. **c** Front view of HZTMEC-3rGO. **d** Top view of HZTMEC-3rGO.


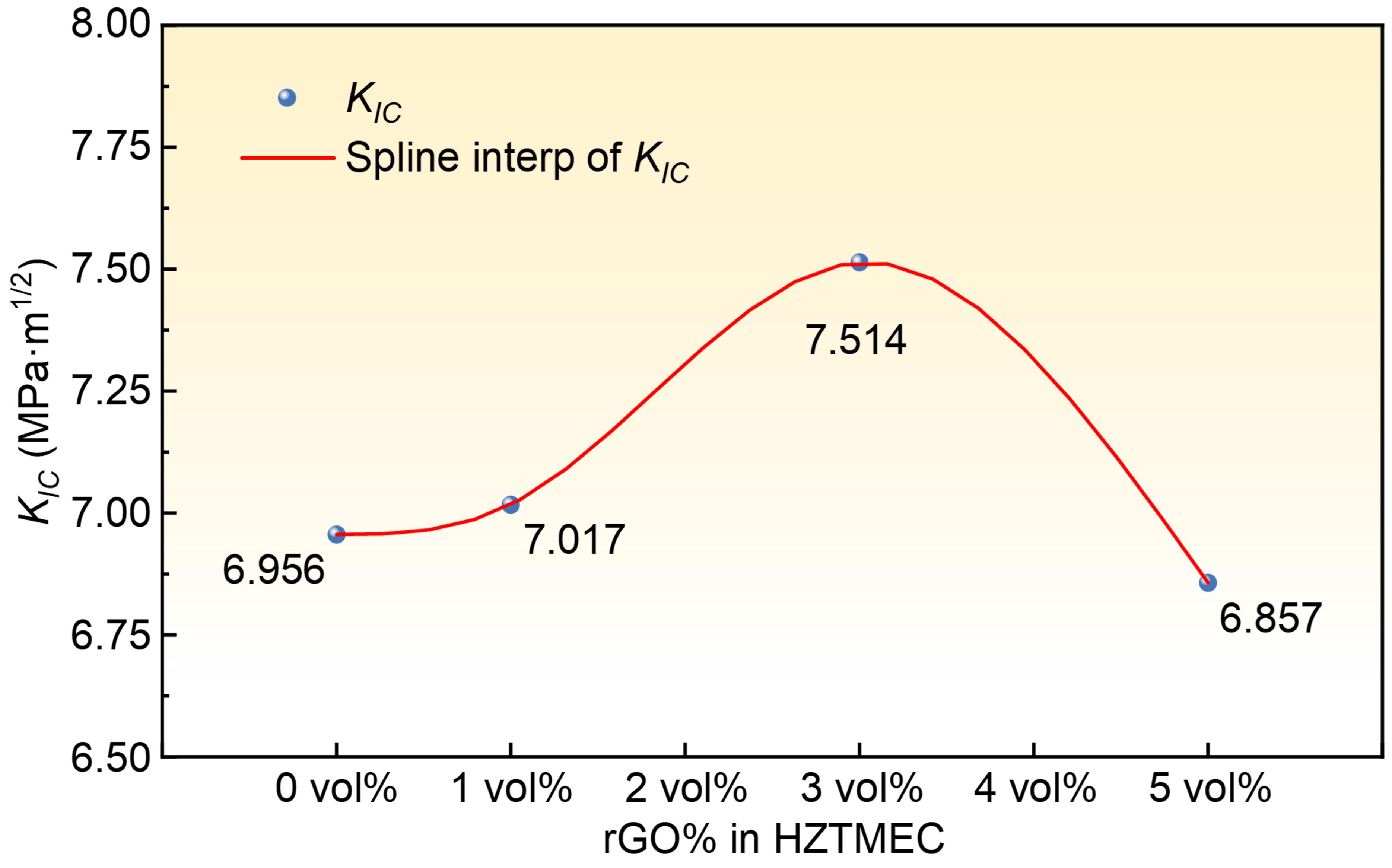


**Figure S11 Cubic spline interpolation based on *K_IC_* data of each sample.**

**
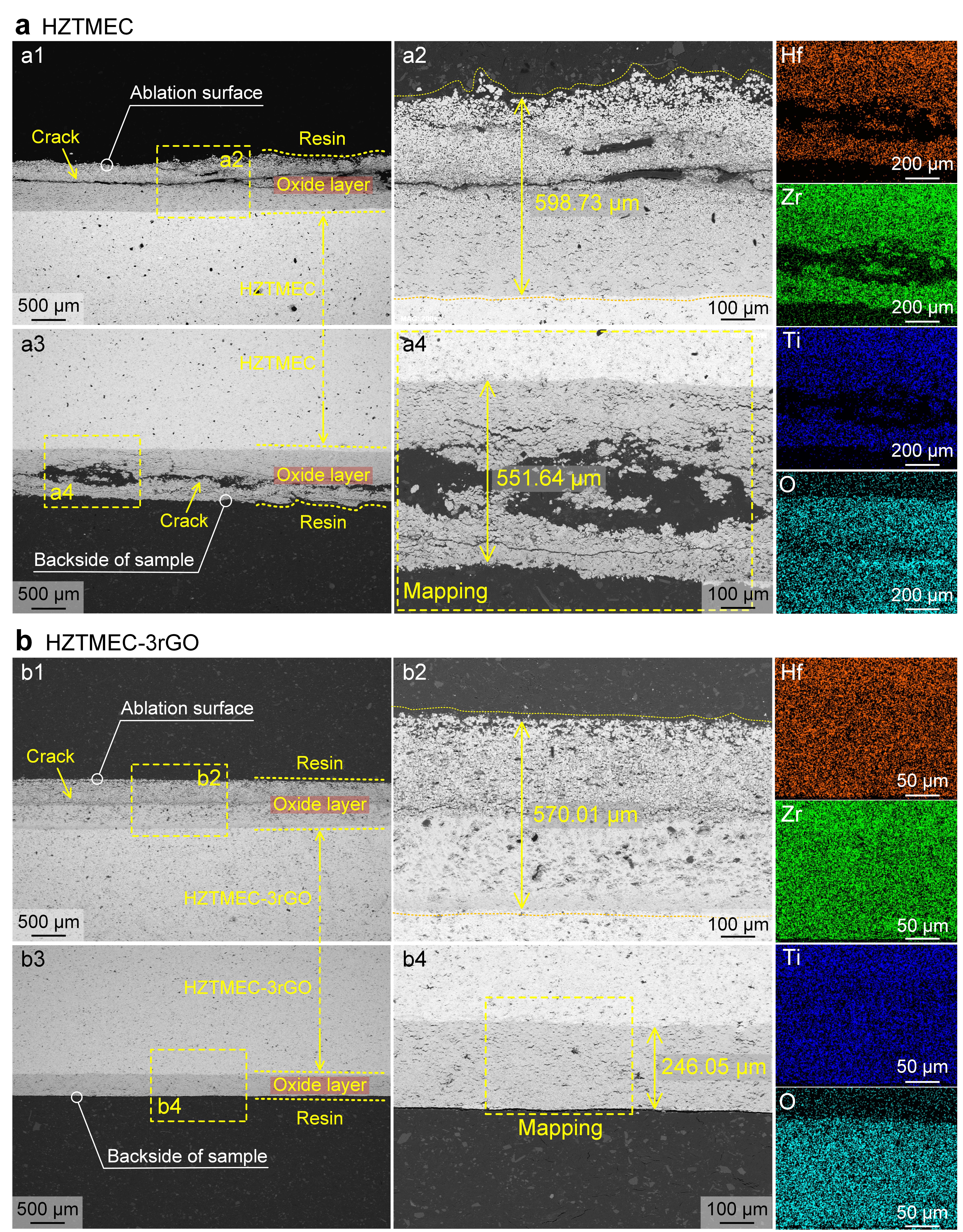
**

**Figure S12** **Cross-sections of the oxide layers. a** HZTMEC: **a1,a2** show the cross-section of the oxide layer at the ablation surface, and **a3,a4** show the cross-section of the oxide layer at the backside of the sample, with corresponding elemental mapping. **b** HZTMEC-3rGO: **b1,b2** show the cross-section of the oxide layer at the ablation surface, and **b3,b4** show the cross-section of the oxide layer at the backside of the sample, with corresponding elemental mapping.

**
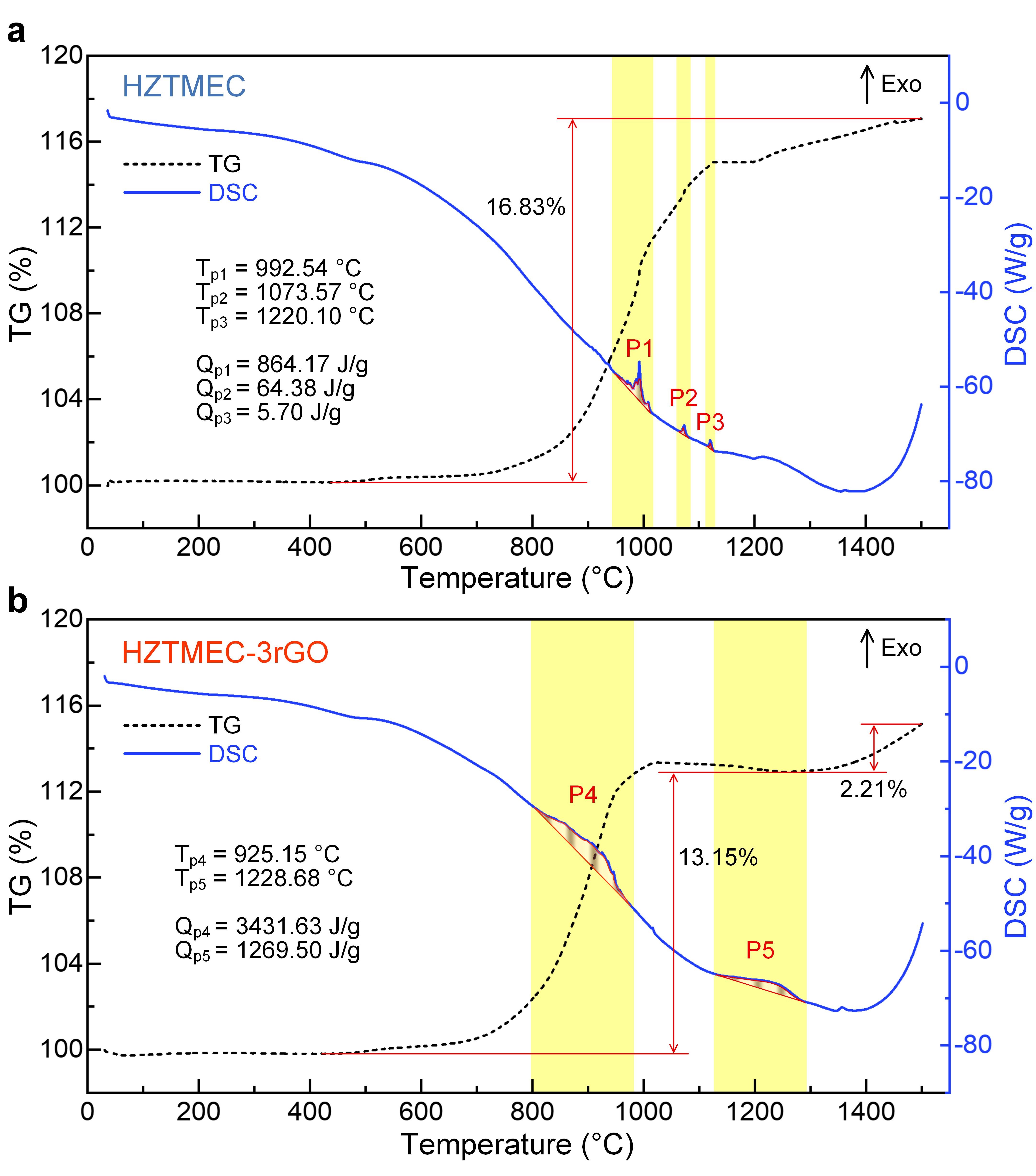
**

**Figure S13** **TG-DSC curves, showing the oxidation behavior of the samples. a** HZTMEC. **b** HZTMEC-3rGO. P1-P5 are the sequence numbers of the exothermic peaks, T is the peak temperature corresponding to each exothermic peak, and Q is the heat release per unit mass corresponding to each exothermic peak.


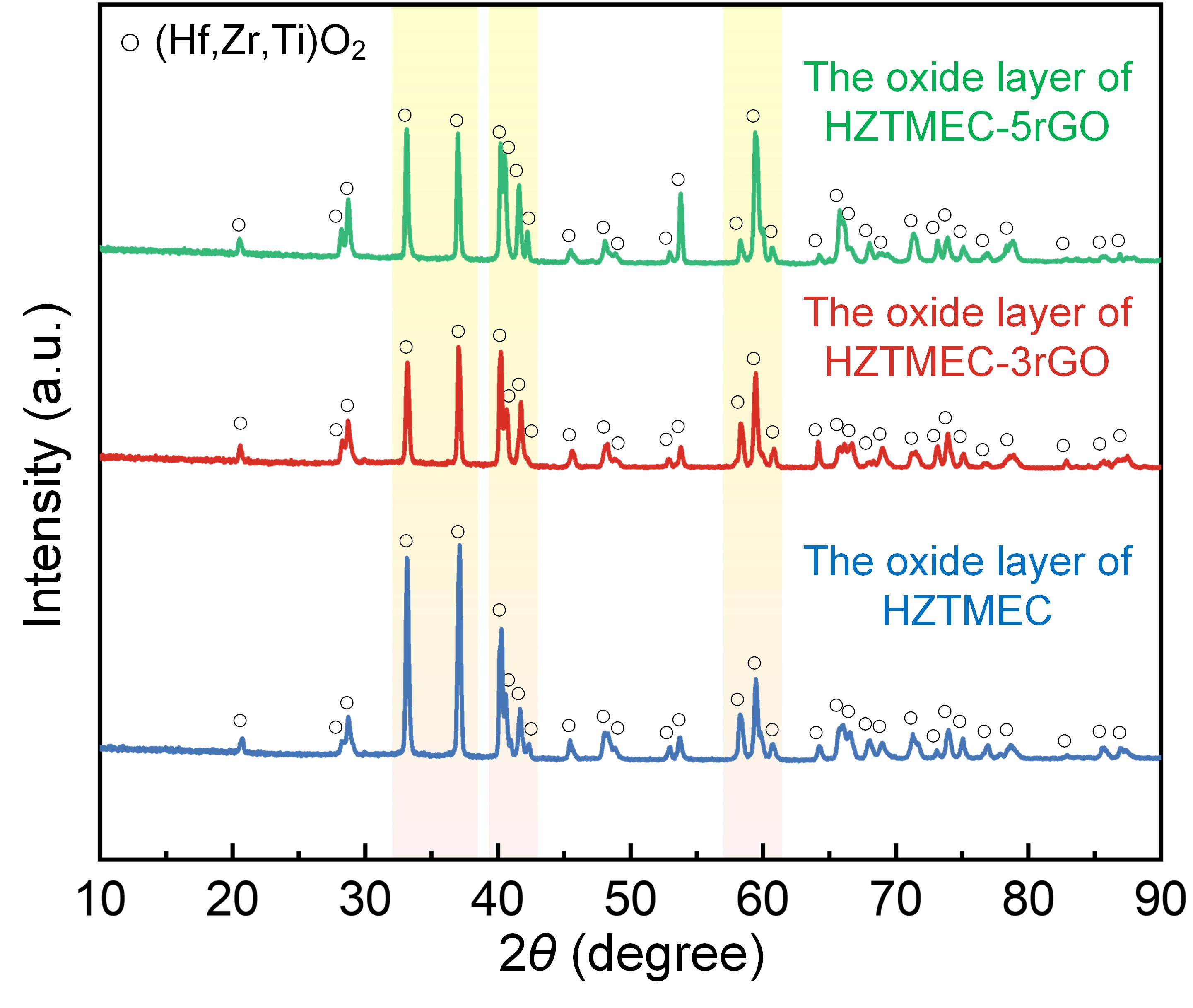


**Figure S14** **The XRD patterns of** **the oxide layers of HZTMEC, HZTMEC-3rGO, and HZTMEC-5rGO all showed the (Hf, Zr, Ti)O_2_ phase.**


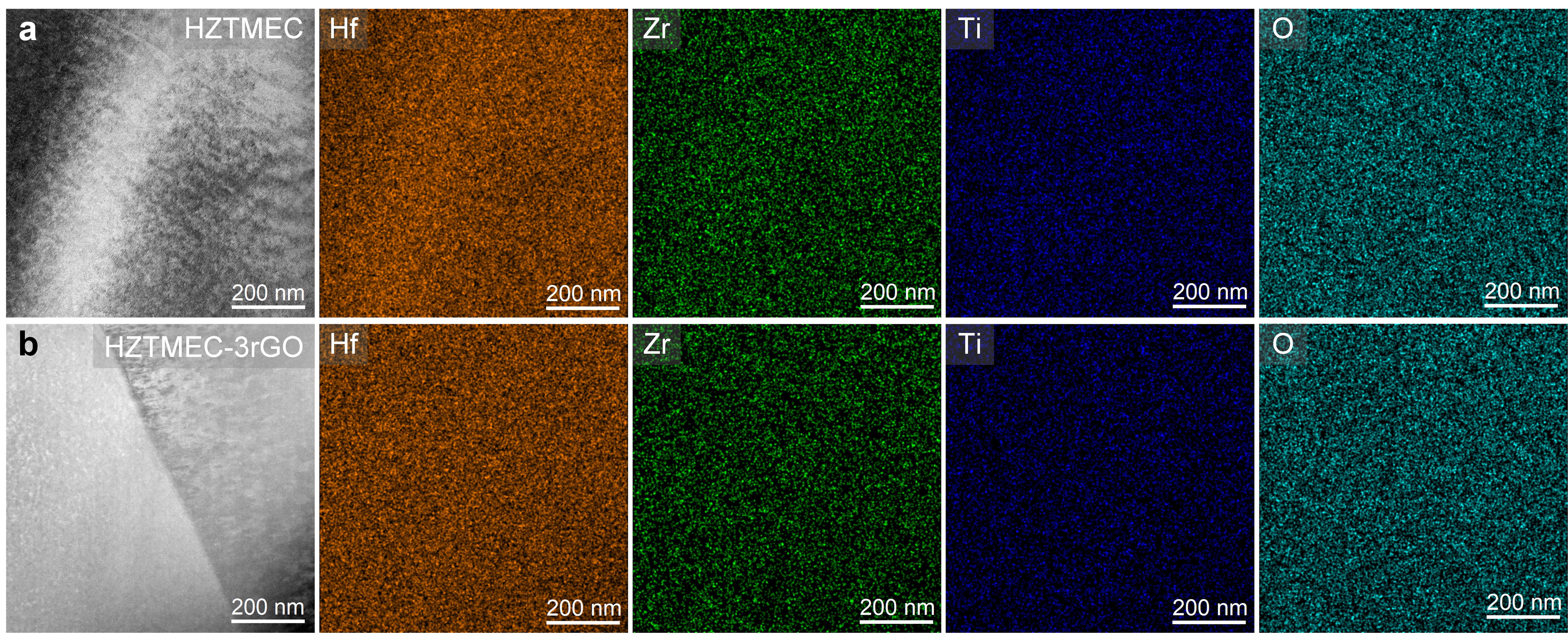


**Figure S15** **HAADF and corresponding elemental mapping** **of the oxide layers. a** The oxide layer of HZTMEC. **b** The oxide layer of HZTMEC-3rGO.


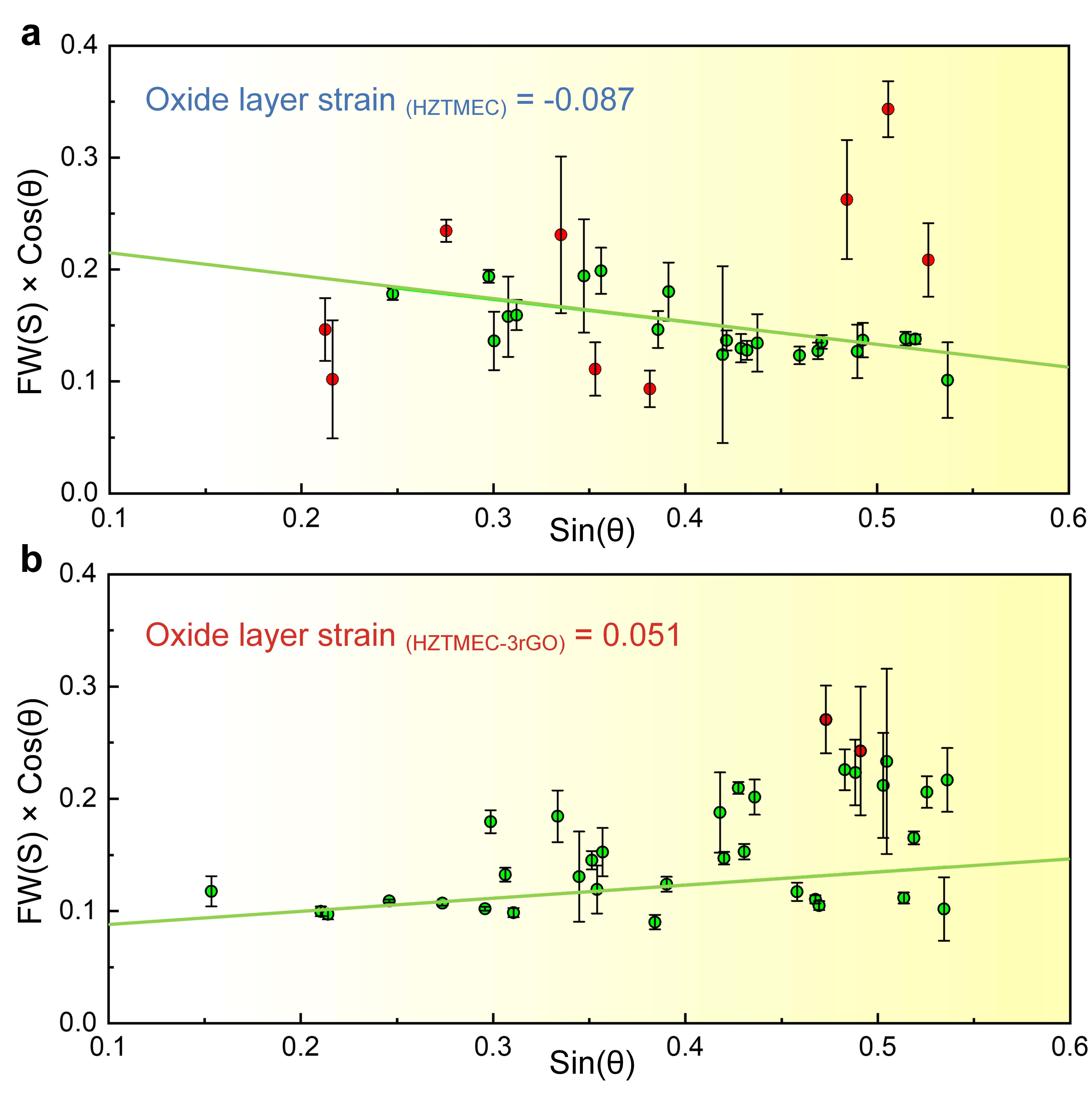


**Figure S16 Strain analysis results of the oxide layer based on the Debye-Scherrer formula.** **a** The oxide layer of HZTMEC. **b** The oxide layer of HZTMEC-3rGO.


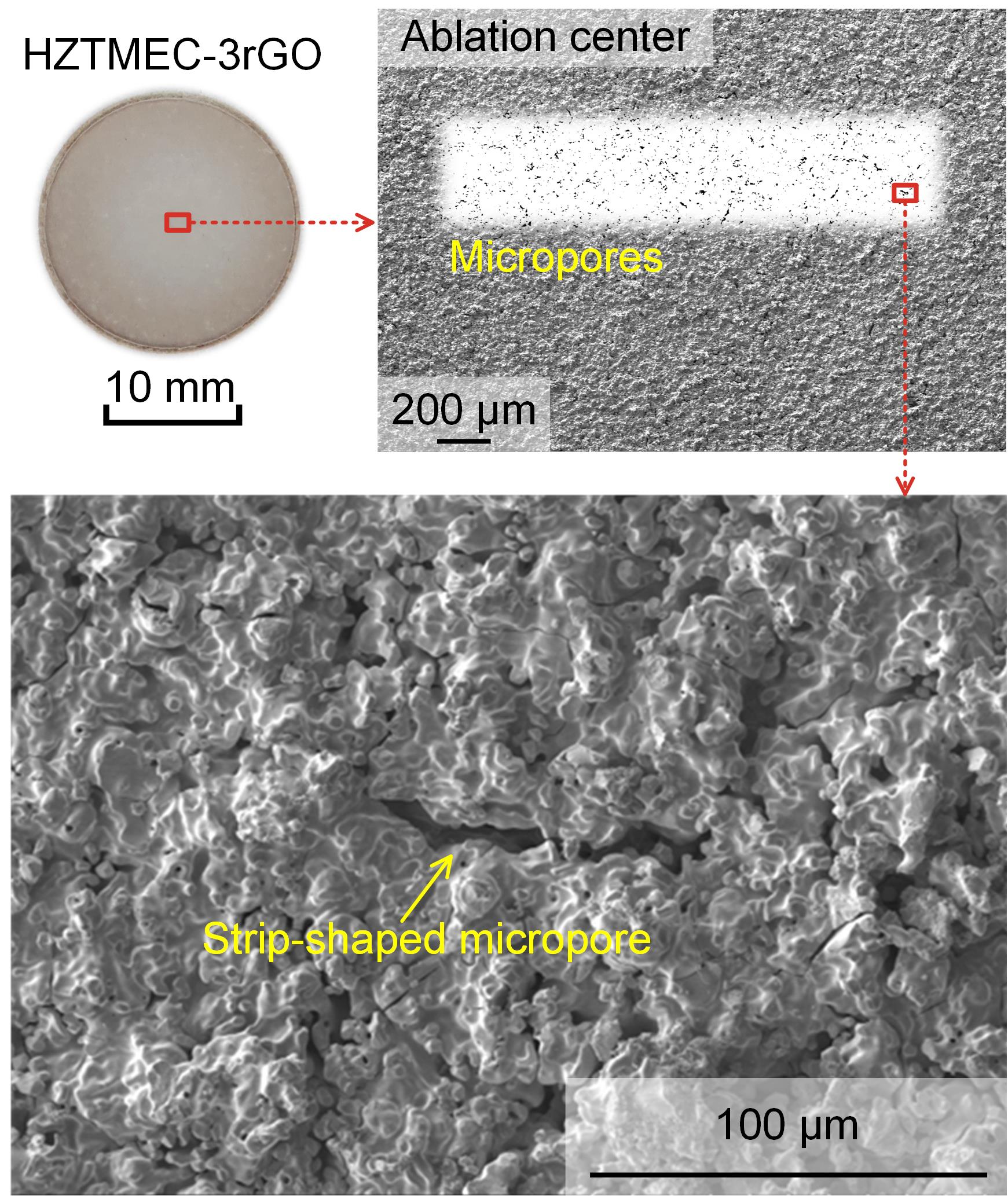


**Figure S17** **Strip-shaped micropores with dimensions similar to those of rGO were observed on the oxide layer surface of HZTMEC-3rGO.**

**

**

**Figure S18** **Finite element simulation results of HZTMEC and HZTMEC-3rGO during the ablation heating period. a** 3D model. **b** Temperature distribution. **c** Equivalent stress distribution. **d** First principal stress distribution.


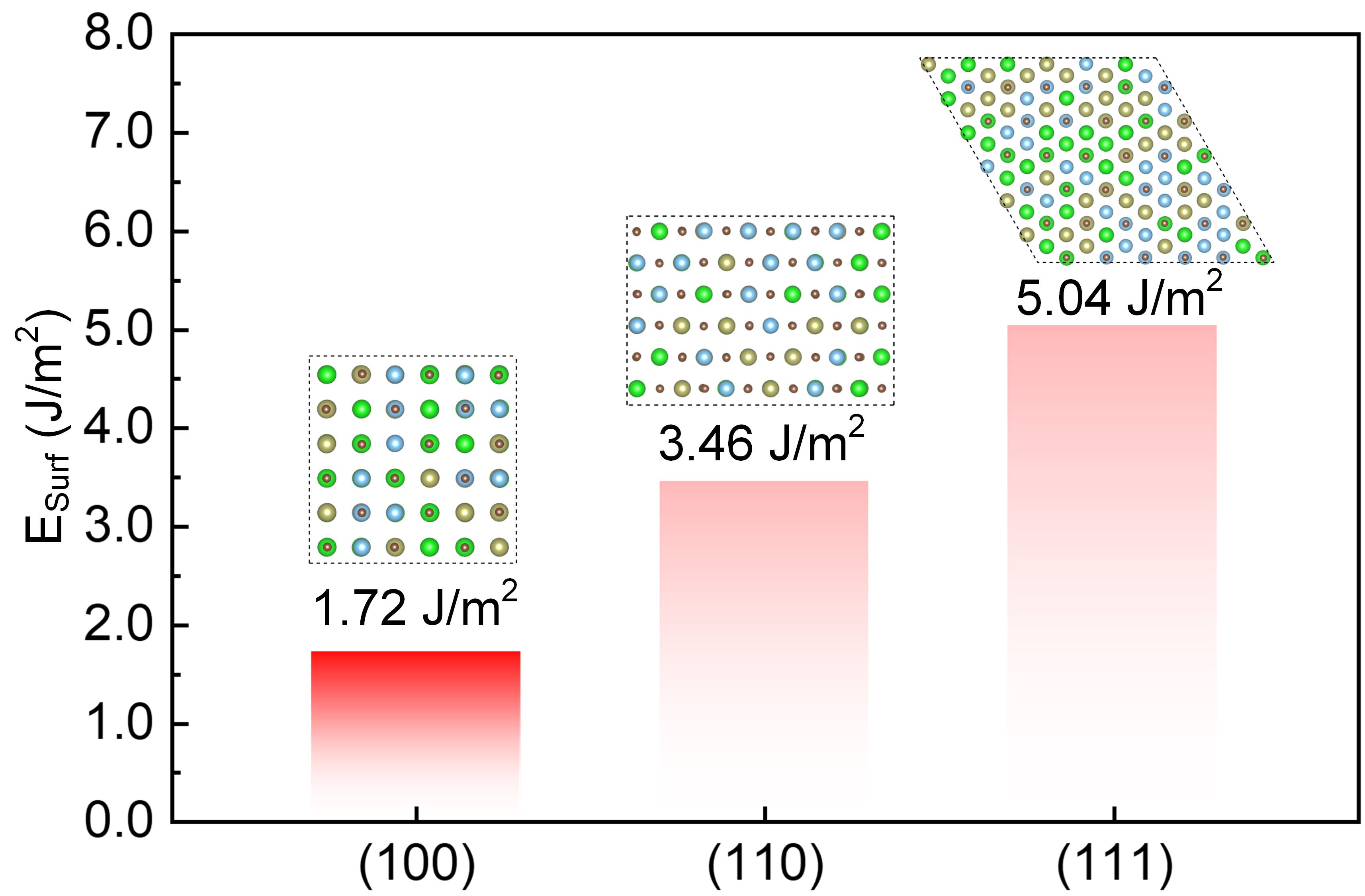


**Figure S19** **Surface formation energy of low-index crystal faces of HZTMEC obtained by first-principles calculations.**

**Supplementary Tables**

**Table S1** **Crystal structure parameters of HZTMEC from** **Rietveld refinement of XRD.**

| (Hf_1/2_Zr_1/3_Ti_1/6_)C | Space group | Unit cell | | | Characteristic peaks | | |
| --- | --- | --- | --- | --- | --- | --- | --- |
|  |  | a (Å)  b(Å)  c(Å) | α(°)  β(°)  γ (°) | Volume (Å^3^) | h k l | d (Å) | Position (°) |
| Crystal structure parameters | *Fmm* | 4.61467  4.61467  4.61467 | 90  90  90 | 98.27 | 1 1 1 | 2.664 | 33.732 |
|  |  |  |  |  | 2 0 0 | 2.307 | 39.126 |
|  |  |  |  |  | 2 2 0 | 1.631 | 56.466 |
|  |  |  |  |  | 3 1 1 | 1.391 | 67.351 |
|  |  |  |  |  | 2 2 2 | 1.332 | 70.773 |
|  |  |  |  |  | 4 0 0 | 1.153 | 83.897 |

**Table S2** **Crystal structure parameters of the oxide layer of HZTMEC-3rGO from Rietveld refinement of XRD.**

| (Hf_1/2_Zr_1/3_Ti_1/6_)O_2_ | Space group | Unit cell | | | Main characteristic peaks | | |
| --- | --- | --- | --- | --- | --- | --- | --- |
|  |  | a (Å)  b(Å)  c(Å) | α(°)  β(°)  γ (°) | Volume (Å^3^) | h k l | d (Å) | Position (°) |
| Crystal structure parameters | *P121/c1* | 5.10951  5.16837  5.29175 | 90  99.155  90 | 137.964 | 1 0 0 | 5.0187 | 17.658 |
|  |  |  |  |  | 0 1 1 | 3.6603 | 24.297 |
|  |  |  |  |  | 1 1 0 | 3.5972 | 24.73 |
|  |  |  |  |  | -1 1 1 | 3.1318 | 28.477 |
|  |  |  |  |  | 1 1 1 | 2.8149 | 31.763 |
|  |  |  |  |  | 0 0 2 | 2.6038 | 34.415 |
|  |  |  |  |  | 0 2 0 | 2.579 | 34.757 |
|  |  |  |  |  | 2 0 0 | 2.5151 | 35.669 |
|  |  |  |  |  | -1 0 2 | 2.4801 | 36.19 |
|  |  |  |  |  | 0 1 2 | 2.31 | 38.959 |
|  |  |  |  |  | 0 2 1 | 2.2346 | 40.329 |
|  |  |  |  |  | 1 2 0 | 2.1929 | 41.13 |
|  |  |  |  |  | 2 1 0 | 2.1758 | 41.468 |
|  |  |  |  |  | -1 1 2 | 2.1584 | 41.818 |
|  |  |  |  |  | -2 1 1 | 2.0055 | 45.174 |

**Table S3 The main parameters of the first-principles calculation and modeling.**

| Calculation contents | Energy cutoff (eV) | Electronic energy convergence criterion (eV) | Ionic force convergence criterion (eV/Å) | Supercell |
| --- | --- | --- | --- | --- |
| Surface formation energy | 500 | 1×10^-5^ | -0.03 | 3×3×3 |
| Oxygen adsorption energy | 500 | 1×10^-5^ | -0.03 | 3×5×1 |

**Table S4** **Attribute parameters of material in finite element simulation.**

| Attribute parameters | Numerical value | Unit |
| --- | --- | --- |
| Density | 9590 | kg/m^3^ |
| Thermal conductivity | 1.72 | W/(m·K) |
| Specific heat | 120 | J/(kg·K) |
| Thermal expansion | 5.6 | 10^-6^/K |
| Young’s modulus | 158.3 | GPa |
| Poisson’s ratio | 0.36 | 1 |
